# Supplementary material for: A novel strategy to generate immunocytokines with activity-on-demand using small molecule inhibitors
Source: EMBO Mol Med. 2024 Mar 6;16(4):18. doi: 10.1038/s44321-024-00034-0 (PMC11018789; doi:10.1038/s44321-024-00034-0)
Supplement: Supplementary file 1 — Appendix [file 44321_2024_34_MOESM1_ESM.pdf]

APPENDIX

A novel strategy to generate immunocytokines with activity-on-demand using small molecule inhibitors

Giulia Rotta[1,2], Ettore Gilardoni[1], Domenico Ravazza[1], Jacqueline Mock[1], Frauke Seehusen[3], Abdullah Elsayed[1,4], Emanuele Puca[1,5], Roberto De Luca[1], Christian Pellegrino[6], Thomas Look[7], Tobias Weiss[7], Markus G. Manz[6], Cornelia Halin[4], Dario Neri[1,5]*, Sheila Dakhel Plaza[1]*

Table of Contents

Appendix Table S1. Aminoacidic sequence of L19-mIL12 2

Appendix Table S2. Aminoacidic sequence of L19-IL12 (clinical product carrying human IL12) 2

Appendix Table S3. Single-concentration external calibration points 4

Appendix Table S4. Statistical analysis of therapy experiments 5

Appendix Figure S1. Representative gating strategy for phenotypic analysis of spleen and blood. 14

Appendix Figure S2. Representative gating strategy for identification of IFN-γ+ cells in MC-38 tumors. 15

Appendix Figure S3. Representative gating strategy for p-STAT4 analysis in blood. 16

Appendix Table S1. Aminoacidic sequence of L19-mIL12

mIL12 p40-Linker-mIL12 p35- Linker-L19-Linker-L19
MWELEKDVYVVEVDWTPDAPGETVNLTCDTPEEDDITWTSDQRHGVIGSGKTLTITVKEFLDAGQYTCHK<br>GGETLSHSHLLLHKKENGIWSTEILKNFKNKTFLKCEAPNYSGRFTCSWLVQRNMDLKFNIKSSSSSPDSR<br>AVTCGMASLSAEKVTLDQRDYEKYSVSCQEDVTCPTAEETLPIELALEARQQNKYENYSTSFFIRDIIKPDP<br>PKNLQMRPLKNSQVEVSWEYPDSWSTPHSYFSLKFFVRIQRKKEKMKETEEGCNQKGAFLVERTSTEVQ<br>CKGGNVCVQAQDRYYNSSCSKWACVPCRVRSGGGGSGGGGSGGGGSRVIPVSGPARCLSQSRNLLK<br>TTDDMVKTAREKLKHYSCTAEDIDHEDITRDQTSTLKTCLPLELHKNESCLATRETSSTTRGSCLPPQKTS<br>LMMTLCLGSIYEDLKMYQTEFQAINAALQNHNHQQIILDKGMLVAIDELMQSLNHNGETLRQKPPVGEADP<br>YRVKMKLCILLHAFSTRVVTINRVMGYLSSAGSADGEVQLLESGGGLVQPGGSLRLSCAASGFTFSSFSM<br>SWVRQAPGKGLEWVSSISGSSGTTYYADSVKGRFTISRDNSKNTLYLQMNSLRAEDTAVYYCAKPFPYF<br>DYWGQGTLVTVSSGSSGGEIVLTQSPGTLSLSPGERATLSCRASQSVSSSFLAWYQQKPGQAPRLLIYY<br>ASSRATGIPDRFSGSGSGTDFTLTISRLEPEDFAVYYCQQTGRIPPTFGQGTKVEIKSSSSGSSSSGSSSS<br>GEVQLLESGGGLVQPGGSLRLSCAASGFTFSSFSMSWVRQAPGKGLEWVSSISGSSGTTYYADSVKGR<br>FTISRDNSKNTLYLQMNSLRAEDTAVYYCAKPFPYFDYWGQGTLVTVSSGSSGGEIVLTQSPGTLSLSPG<br>ERATLSCRASQSVSSSFLAWYQQKPGQAPRLLIYYASSRATGIPDRFSGSGSGTDFTLTISRLEPEDFAVY<br>YCQQTGRIPPTFGQGTKVEIK

Appendix Table S2. Aminoacidic sequence of L19-IL12 (clinical product carrying human IL12)

IL12 p40-Linker-IL12 p35- Linker-L19-Linker-L19
IWELKKDVYVVELDWYPDAPGEMVVLTCDTPEEDGITWTLDQSSEVLGSGKTLTIQVKEFGDAGQYTCHK<br>GGEVLSHSLLLLHKKEDGIWSTDILKDQKEPKNKTFLRCEAKNYSGRFTCWWLTTISTDLTFSVKSSRGSS<br>DPQGVTCGAATLSAERVRGDNKEYEYSVECQEDSACPAAEESLPIEVMVDAVHKLKYENYTSSFFIRDIIK<br>PDPPKNLQLKPLKNSRQVEVSWEYPDTWSTPHSYFSLTFCVQVQGKSKREKKDRVFTDKTSATVICRKN<br>ASISVRAQDRYYSSSWSEWASVPCSGGGGSGGGGSGGGGSRNLPVATPDPGMFPCLHHSQNLLRAVS<br>NMLQKARQTLEFYPCTSEEIDHEDITKDKTSTVEACLPLELTKNESCLNSRETSFITNGSCLASRKTSFMM<br>ALCLSSIYEDLKMYQVEFKTMNAKLLMDPKRQIFLDQNMLAVIDELMQALNFNSETVPQKSSLEEPDFYKT<br>KIKLCILLHAFRIRAVTIDRVMSYLNASGSADGGSSAGGSDAGEVQLLESGGGLVQPGGSLRLSCAASGFT

FSSFSMSWVRQAPGKGLEWVSSISGSSGTTYYADSVKGRFTISRDNSKNTLYLQMNSLRAEDTAVYYCA
KPFPYFDYWGQGTLVTVSSGSSGGEIVLTQSPGTLSLSPGERATLSCRASQSVSSSFLAWYQQKPGQAP
RLLIYYASSRATGIPDRFSGSGSGTDFTLTISRLEPEDFAVYYCQQTGRIPPTFGQGTKVEIKSSSSGSSSS
GSSSSGEVQLLESGGGLVQPGGSLRLSCAASGFTFSSFSMSWVRQAPGKGLEWVSSISGSSGTTYYAD
SVKGRFTISRDNSKNTLYLQMNSLRAEDTAVYYCAKPFPYFDYWGQGTLVTVSSGSSGGEIVLTQSPGTL
SLSPGERATLSCRASQSVSSSFLAWYQQKPGQAPRLLIYYASSRATGIPDRFSGSGSGTDFTLTISRLEPE
DFAVYYCQQTGRIPPTFGQGTKVEIK

Appendix Table S3. Single-concentration external calibration points

Organ	pmol injected in the MS	Ratio with Internal standard
Serum	1	0.64 ± 0.03
Tumor	1	0.65 ± 0.02
Liver	1	0.64 ± 0.01
Spleen	1	0.67 ± 0.03

Appendix Table S3: Ratios in each organ between extrapolated MS areas of fixed amounts of Ruxolitinib and internal standard were calculated and used as external single calibration points for quantification of the biodistribution experiments. Values are expressed as mean ± SD, n = 4.

Appendix Table S4. Statistical analysis of therapy experiments

Differences in tumor volume and body weight between therapeutic groups were compared using the two-way ANOVA analysis with Bonferroni post-test of GraphPad Prism 7 (La Jolla, CA, USA). Survival curves were compared by Mantel-Cox test. Days are counted after tumor implantation.

Tumor volume (mm³)

Fig. 2a

Saline vs Ruxolitinib (75 mg/kg)

From day 7 to day 15	non-significant differences

Saline vs L19-mIL12 (0.6 mg/kg)

From day 7 to day 10	non-significant difference
From day 11 to day 15	$p < 0.0001$

Saline vs Ruxolitinib (75 mg/kg) + L19-mIL12 (0.6 mg/kg)

From day 7 to day 9	non-significant difference
day 10	$p < 0.01$
From day 11 to day 15	$p < 0.0001$

Ruxolitinib (75 mg/kg) vs L19-mIL12 (0.6 mg/kg)

From day 7 to day 11	non-significant difference
day 12	$p < 0.05$
From day 13 to day 15	$p < 0.0001$

Ruxolitinib (75 mg/kg) vs Ruxolitinib (75 mg/kg) + L19-mIL12 (0.6 mg/kg)

From day 7 to day 11	non-significant difference
day 12	$p < 0.01$
From day 13 to day 15	$p < 0.0001$

L19-mIL12 (0.6 mg/kg) vs Ruxolitinib (75 mg/kg) + L19-mIL12 (0.6 mg/kg)

From day 7 to day 26	non-significant difference

Fig. 2b

Saline vs L19-mIL12 (0.9 mg/kg)

From day 7 to day 10	non-significant difference
From day 11 to day 12	$p < 0.001$
From day 13 to day 15	$p < 0.0001$

Saline vs Ruxolitinib (75 mg/kg) + L19-mIL12 (0.9 mg/kg)

From day 7 to day 9	non-significant difference
day 10	$p < 0.05$
From day 11 to day 15	$p < 0.0001$

Ruxolitinib (75 mg/kg) vs L19-mIL12 (0.9 mg/kg)

From day 7 to day 11	non-significant difference
day 12	$p < 0.05$
day 13	$p < 0.01$
From day 14 to day 15	$p < 0.0001$

Ruxolitinib (75 mg/kg) vs Ruxolitinib (75 mg/kg) + L19-mIL12 (0.9 mg/kg)

From day 7 to day 11	non-significant difference
day 12	$p < 0.01$
From day 13 to day 15	$p < 0.0001$

L19-mIL12 (0.9 mg/kg) vs Ruxolitinib (75 mg/kg) + L19-mIL12 (0.9 mg/kg)

From day 7 to day 26	non-significant difference

Fig. 2c

Saline vs L19-mIL12 (1.2 mg/kg)

From day 7 to day 10	non-significant difference
From day 11 to day 12	$p < 0.001$
From day 13 to day 15	$p < 0.0001$

Saline vs Ruxolitinib (75 mg/kg) + L19-mIL12 (1.2 mg/kg)

From day 7 to day 9	non-significant difference
day 10	$p < 0.05$
From day 11 to day 15	$p < 0.0001$

Ruxolitinib (75 mg/kg) vs L19-mIL12 (1.2 mg/kg)

From day 7 to day 11	non-significant difference
day 12	$p < 0.05$
From day 13 to day 15	$p < 0.0001$

Ruxolitinib (75 mg/kg) vs Ruxolitinib (75 mg/kg) + L19-mIL12 (1.2 mg/kg)

From day 7 to day 11	non-significant difference
day 12	$p < 0.01$
From day 13 to day 15	$p < 0.0001$

L19-mIL12 (0.9 mg/kg) vs Ruxolitinib (75 mg/kg) + L19-mIL12 (1.2 mg/kg)

From day 7 to day 26	non-significant difference

<u>Fig. 2g</u>

Saline vs Ruxolitinib (75 mg/kg)

From day 5 to day 13	non-significant difference

Saline vs L19-mIL12 (2.4 mg/kg)

day 5	non-significant difference
day 6	$p < 0.05$
From day 7 to day 10	$p < 0.0001$

Saline vs Ruxolitinib (75 mg/kg) + L19-mIL12 (2.4 mg/kg)

day 5	non-significant
day 6	$p < 0.05$
day 7	$p < 0.01$

From day 8 to day 13	$p < 0.0001$

Ruxolitinib (75 mg/kg) vs L19-mIL12 (2.4 mg/kg)

From day 5 to day 6	non-significant difference
day 7	$p < 0.01$
From day 8 to day 10	$p < 0.0001$

Ruxolitinib (75 mg/kg) vs Ruxolitinib (75 mg/kg) + L19-mIL12 (2.4 mg/kg)

From day 5 to day 7	non-significant difference
From day 8 to day 13	$p < 0.0001$

L19-mIL12 (0.9 mg/kg) vs Ruxolitinib (75 mg/kg) + L19-mIL12 (2.4 mg/kg)

From day 5 to day 10	non-significant difference

<u>Fig. 6a</u>

Saline vs Ruxolitinib (75 mg/kg)

From day 6 to day 14	non-significant difference

Saline vs L19-mIL12 (1.2 mg/kg)

From day 6 to day 8	non-significant difference
day 9	$p < 0.05$
From day 10 to day 14	$p < 0.0001$

Saline vs Ruxolitinib (75 mg/kg) + L19-mIL12 (1.2 mg/kg)

From day 6 to day 9	non-significant difference
From day 10 to day 14	$p < 0.0001$

Ruxolitinib (75 mg/kg) vs L19-mIL12 (1.2 mg/kg)

From day 6 to day 9	non-significant difference
day 10	$p < 0.05$
day 11	$p < 0.001$

From day 12 to day 14	$p < 0.0001$

Ruxolitinib (75 mg/kg) vs Ruxolitinib (75 mg/kg) + L19-mIL12 (1.2 mg/kg)

From day 6 to day 10	non-significant difference
day 11	$p < 0.01$
From day 12 to day 14	$p < 0.0001$

L19-mIL12 (0.9 mg/kg) vs Ruxolitinib (75 mg/kg) + L19-mIL12 (1.2 mg/kg)

From day 6 to day 26	non-significant difference

Body Weight Change (%)

<u>Fig. 2d</u>

Saline vs Ruxolitinib (75 mg/kg)

From day 7 to day 15	non-significant differences

Saline vs L19-mIL12 (0.6 mg/kg)

From day 7 to day 11	non-significant differences
day 12	$p < 0.05$
From day 13 to day 15	$p < 0.0001$

Saline vs Ruxolitinib (75 mg/kg) + L19-mIL12 (0.6 mg/kg)

From day 7 to day 15	non-significant differences

Ruxolitinib (75 mg/kg) vs L19-mIL12 (0.6 mg/kg)

From day 7 to day 15	non-significant differences

Ruxolitinib (75 mg/kg) vs Ruxolitinib (75 mg/kg) + L19-mIL12 (0.6 mg/kg).

From day 7 to day 15	non-significant differences

L19-mIL12 (0.6 mg/kg) vs Ruxolitinib (75 mg/Kg) + L19-mIL12 (0.6 mg/Kg).

From day 7 to day 13	non-significant differences

day 14	$p < 0.05$
From day 15 to day 17	$p < 0.001$
day 18	$p < 0.01$
day 19	$p < 0.05$
From day 20	non-significant differences

Fig. 2e

Saline vs L19-mIL12 (0.9 mg/kg)

From day 7 to day 12	non-significant differences
day 13	$p < 0.001$
From day 14 to day 15	$p < 0.0001$

Saline vs Ruxolitinib (75 mg/kg) + L19-mIL12 (0.9 mg/kg)

From day 7 to day 12	non-significant differences
day 13	$p < 0.01$
From day 14 to day 15	non-significant differences

Ruxolitinib (75 mg/kg) vs L19-mIL12 (0.9 mg/kg)

From day 7 to day 15	non-significant difference

Ruxolitinib (75 mg/kg) vs Ruxolitinib (75 mg/kg) + L19-mIL12 (0.9 mg/kg)

From day 7 to day 15	non-significant difference

L19-mIL12 (0.9 mg/kg) vs Ruxolitinib (75 mg/kg) + L19-mIL12 (0.9 mg/kg)

From day 7 to day 16	non-significant difference
day 17	$p < 0.05$
day 18	$p < 0.01$
day 19	$p < 0.05$
From day 20	non-significant difference

Fig. 2f

Saline vs L19-mIL12 (1.2 mg/kg)

From day 7 to day 12	non-significant difference
day 13	$p < 0.001$
From day 14 to day 15	$p < 0.0001$

Saline vs Ruxolitinib (75 mg/kg) + L19-mIL12 (1.2 mg/kg)

From day 7 to day 14	non-significant difference
day 15	$p < 0.001$

Ruxolitinib (75 mg/kg) vs L19-mIL12 (1.2 mg/kg)

From day 7 to day 15	non-significant difference

Ruxolitinib (75 mg/kg) vs Ruxolitinib (75 mg/kg) + L19-mIL12 (1.2 mg/kg)

From day 7 to day 15	non-significant difference

L19-mIL12 (1.2 mg/kg) vs Ruxolitinib (75 mg/kg) + L19-mIL12 (1.2 mg/kg)

From day 7 to day 14	non-significant difference
From day 15 to day 16	$p < 0.05$
From day 17	non-significant difference

<u>Fig. 2h</u>

Saline vs Ruxolitinib (75 mg/kg)

From day 5 to day 13	non-significant difference

Saline vs L19-mIL12 (2.4 mg/kg)

From day 5 to day 7	non-significant difference
From day 8 to day 10	$p < 0.0001$

Saline vs Ruxolitinib (75 mg/kg) + L19-mIL12 (2.4 mg/kg)

From day 5 to day 7	non-significant difference
day 8	$p < 0.05$
From day 9 to day 10	$p < 0.01$

From day 11 to day 13	$p < 0.0001$

Ruxolitinib (75 mg/kg) vs L19-mIL12 (2.4 mg/kg)

From day 5 to day 7	non-significant difference
From day 8 to day 10	$p < 0.0001$

Ruxolitinib (75 mg/kg) vs Ruxolitinib (75 mg/kg) + L19-mIL12 (2.4 mg/kg)

From day 5 to day 9	non-significant difference
day 10	$p < 0.05$
From day 11 to day 13	$p < 0.0001$

L19-mIL12 (0.6 mg/kg) vs Ruxolitinib (75 mg/kg) + L19-mIL12 (2.4 mg/kg)

From day 5 to day 7	non-significant difference
day 8	$p < 0.001$
From day 9 to day 10	$p < 0.0001$

<u>Fig. 6b</u>

Saline vs Ruxolitinib (75 mg/kg)

From day 6 to day 11	non-significant difference
From day 12 to day 13	$p < 0.001$
Day 14	non-significant difference

Saline vs L19-mIL12 (1.2 mg/kg)

From day 6 to day 10	non-significant difference
From day 11 to day 14	$p < 0.0001$

Saline vs Ruxolitinib (75 mg/kg) + L19-mIL12 (1.2 mg/kg)

From day 6 to day 14	non-significant difference

Ruxolitinib (75 mg/kg) vs L19-mIL12 (1.2 mg/kg)

From day 6 to day 9	non-significant difference

day 10	$p < 0.001$
From day 11 to day 14	$p < 0.0001$

Ruxolitinib (75 mg/kg) vs Ruxolitinib (75 mg/kg) + L19-mIL12 (1.2 mg/kg)

From day 6 to day 11	non-significant difference
day 12	$p < 0.001$
day 13	$p < 0.0001$
day 14	non-significant difference

L19-mIL12 (1.2 mg/kg) vs Ruxolitinib (75 mg/kg) + L19-mIL12 (1.2 mg/kg)

From day 6 to day 10	non-significant difference
day 11	$p < 0.05$
day 12	$p < 0.001$
From day 13 to day 14	$p < 0.0001$
day 15	$p < 0.01$
From day 16	non-significant difference

Survival curves

Saline vs Ruxolitinib (75 mg/kg) → non-significant difference

Saline vs L19-mIL12 (2.4 mg/kg) → $p < 0.05$

Saline vs Ruxolitinib (75 mg/kg) + L19-mIL12 (2.4 mg/kg) → $p < 0.01$

Ruxolitinib (75 mg/kg) vs L19-mIL12 (2.4 mg/kg) → $p < 0.01$

Ruxolitinib (75 mg/kg) vs Ruxolitinib (75 mg/kg) + L19-mIL12 (2.4 mg/kg) → $p < 0.01$

L19-mIL12 (2.4 mg/kg) vs Ruxolitinib (75 mg/kg) + L19-mIL12 (2.4 mg/kg) → $p < 0.01$

Appendix Figure S1. Representative gating strategy for phenotypic analysis of spleen and blood.

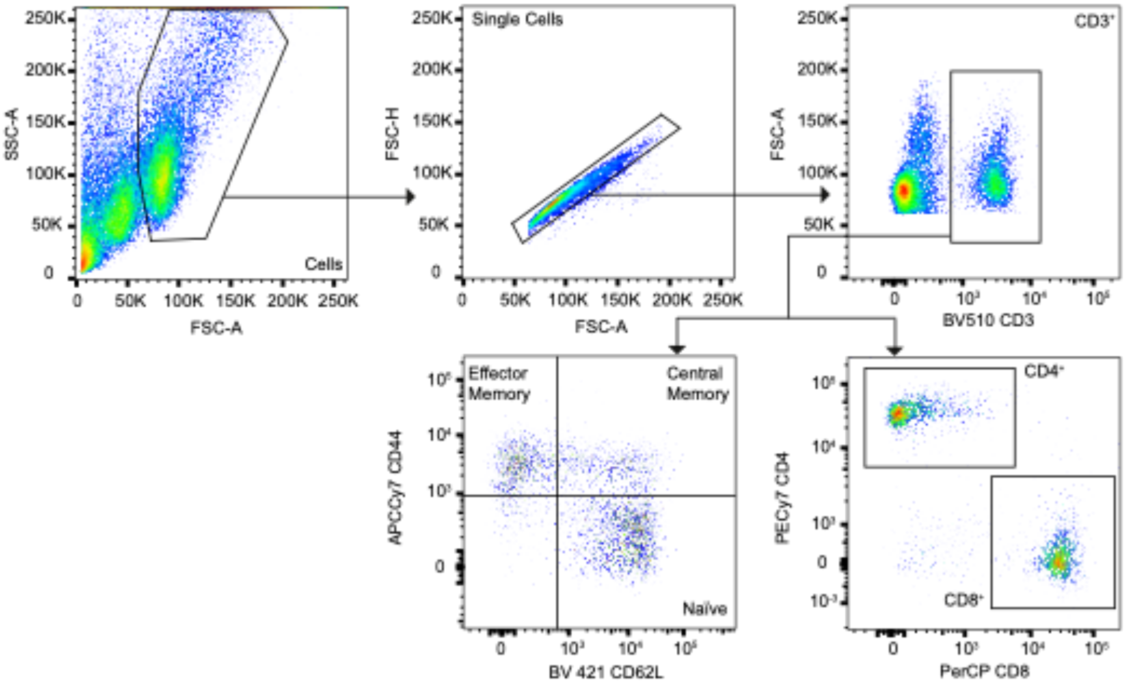

Appendix Figure S1: Representative gating strategy for phenotypic analysis of spleen and blood. Gating was performed on the physical parameters forward scatter area (FSC-A) and side scatter area (SSC-A), followed by identification of singlets. Single cells were gated for expression of CD3 (BV510). CD3+ cells were further gated based on the expression of CD4 (PECy7) and CD8 (PerCP) markers and the expression of differentiation markers CD44 (APCCy7) and CD62L (BV421).

Appendix Figure S2. Representative gating strategy for identification of IFN-γ+ cells in MC-38 tumors.

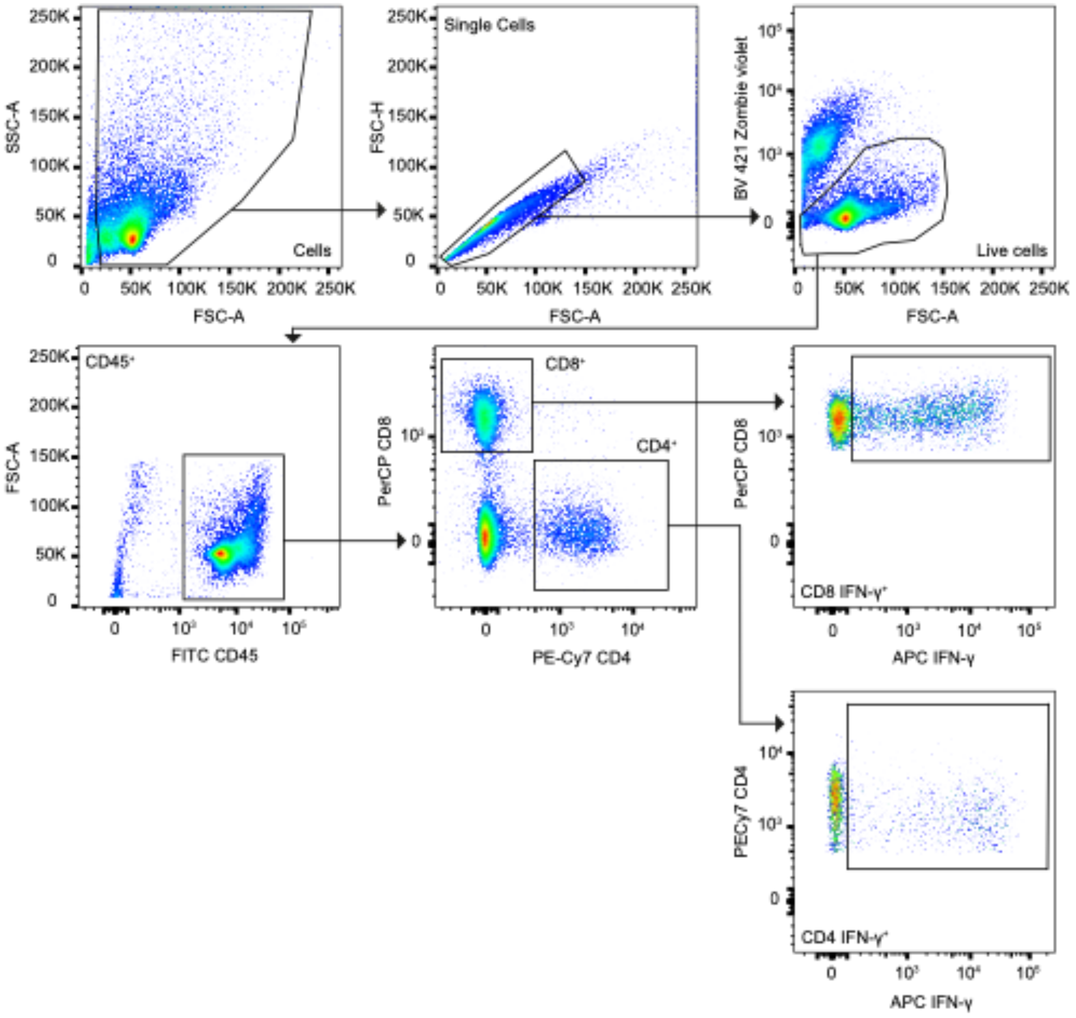

Appendix Figure S2: Representative gating strategy for identification of IFN-γ+ cells in MC-38 tumors. Gating was performed on the physical parameters forward scatter area (FSC-A) and side scatter area (SSC-A), followed by identification of singlets. Live cell discrimination was performed with Zombie Violet (BV421). Immune cells were identified using the CD45 (FITC) expression marker. Gating on CD4+ (PECy7) and CD8+ (PerCP) T cells was followed by analysis of IFN-γ expression (APC).

Appendix Figure S3. Representative gating strategy for p-STAT4 analysis in blood.

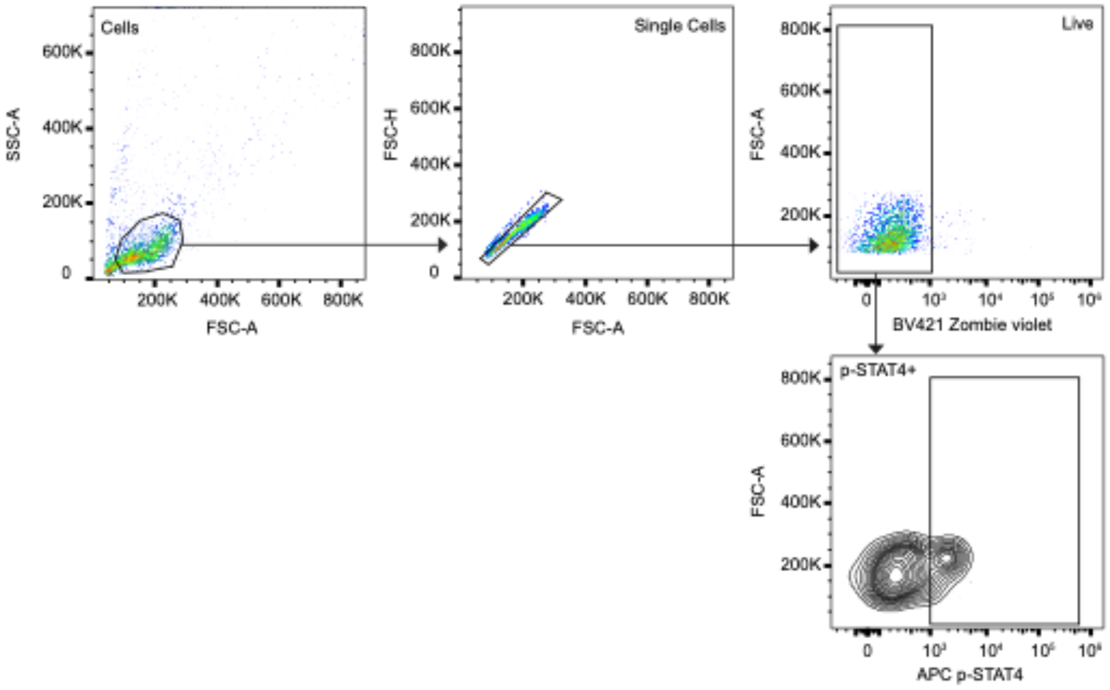

Appendix Figure S3: Representative gating strategy for p-STAT4 analysis in blood. Gating was performed on the physical parameters forward scatter area (FSC-A) and side scatter area (SSC-A), followed by identification of singlets. Live cell discrimination was performed with Zombie Violet (BV421). Phospho-STAT4+ cells (APC) were evaluated within the live cell population.
