# Supplementary figures and images for: A novel strategy to generate immunocytokines with activity-on-demand using small molecule inhibitors
Source: EMBO Mol Med. 2024 Mar 6;16(4):18. doi: 10.1038/s44321-024-00034-0 (PMC11018789; doi:10.1038/s44321-024-00034-0)

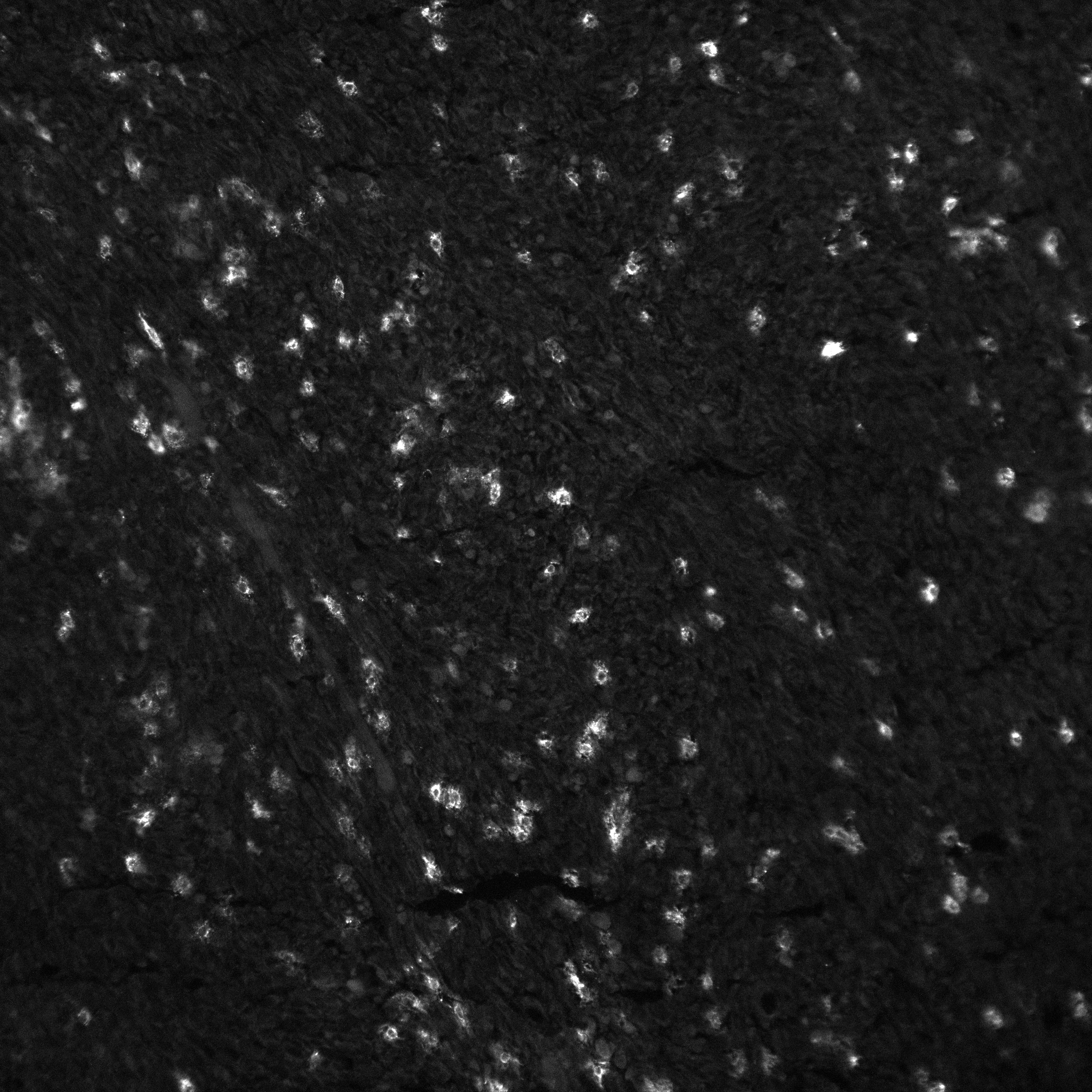

Supplement: Supplementary file 4 — Source Data Fig. 3 [file 44321_2024_34_MOESM4_ESM.zip › Figure 3/3A/Ruxo + L19-mIL12_CD8_10x_bar 100┬╡m.tif]

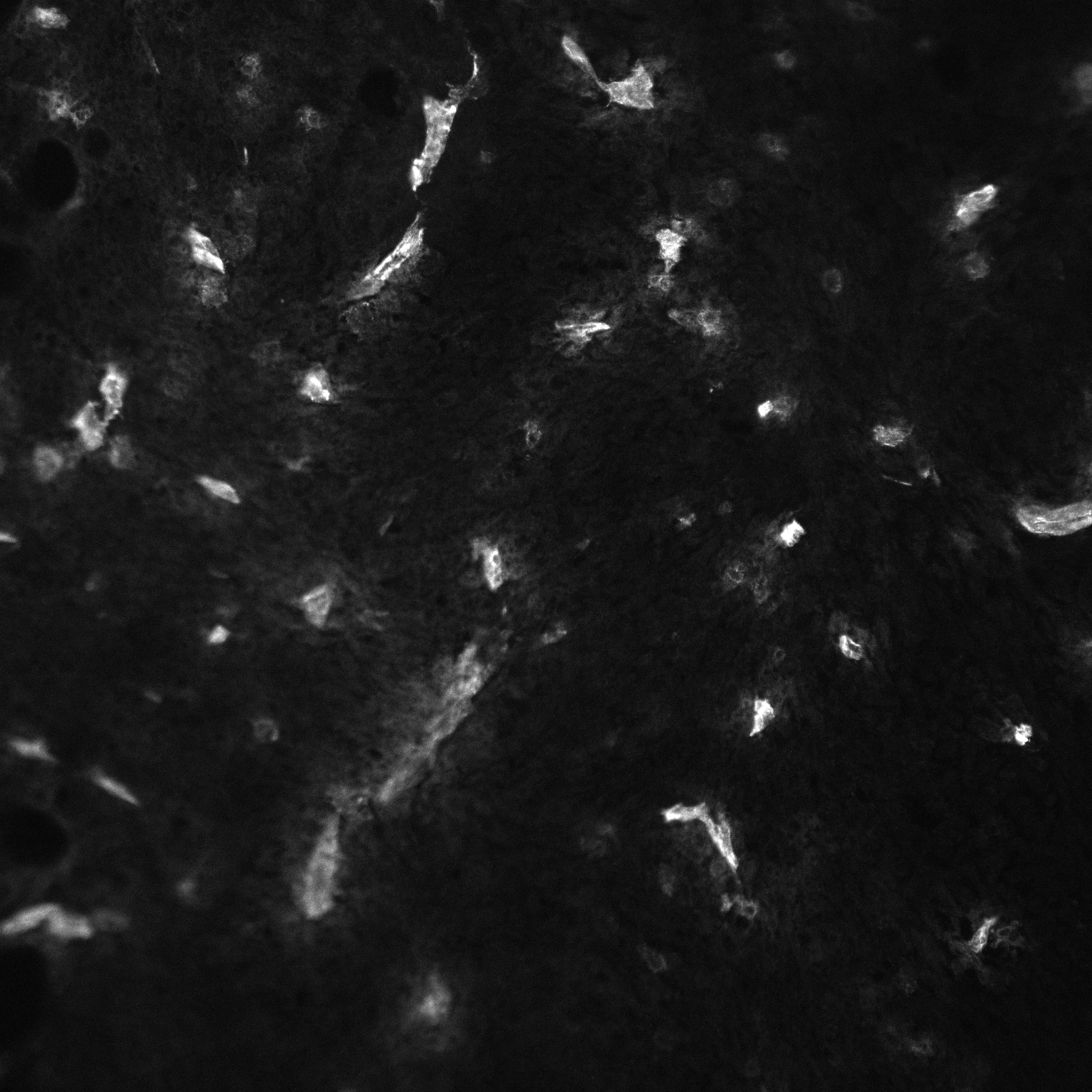

Supplement: Supplementary file 4 — Source Data Fig. 3 [file 44321_2024_34_MOESM4_ESM.zip › Figure 3/3A/L19-mIL12_CD4_10x_bar 100┬╡m.tif]

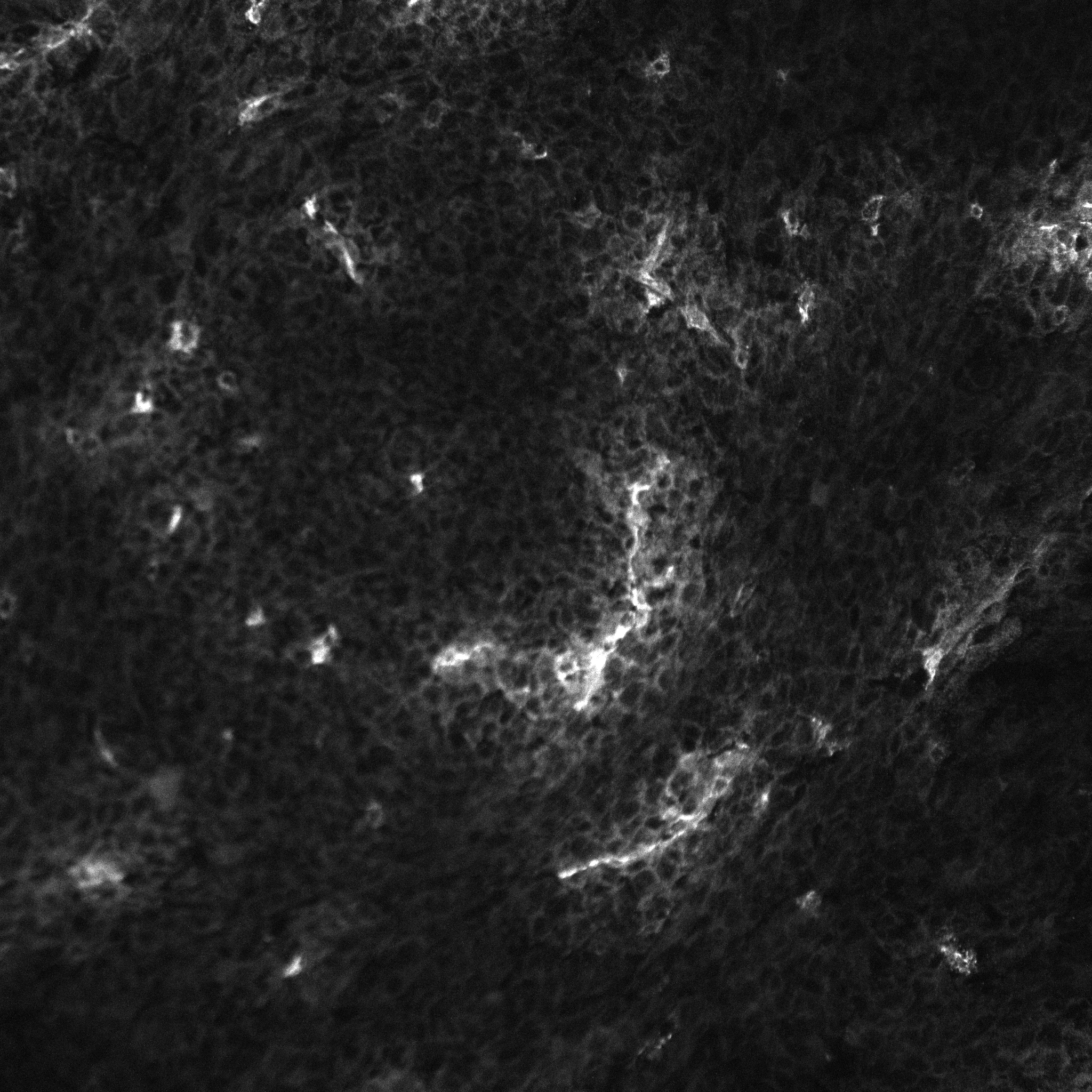

Supplement: Supplementary file 4 — Source Data Fig. 3 [file 44321_2024_34_MOESM4_ESM.zip › Figure 3/3A/Saline_CD4_10x_bar 100┬╡m.tif]

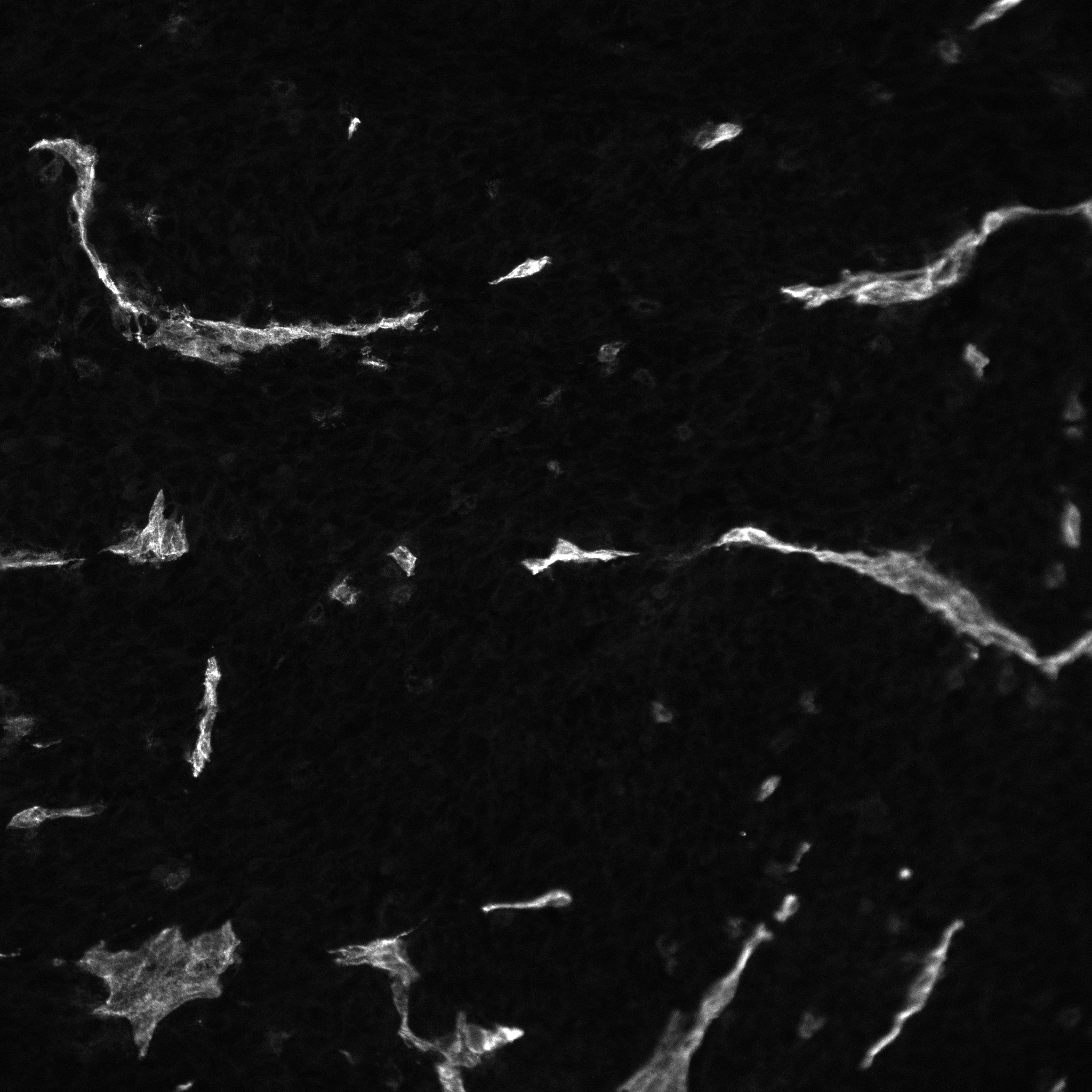

Supplement: Supplementary file 4 — Source Data Fig. 3 [file 44321_2024_34_MOESM4_ESM.zip › Figure 3/3A/Ruxolitinib_CD8_10x_bar 100┬╡m.tif]

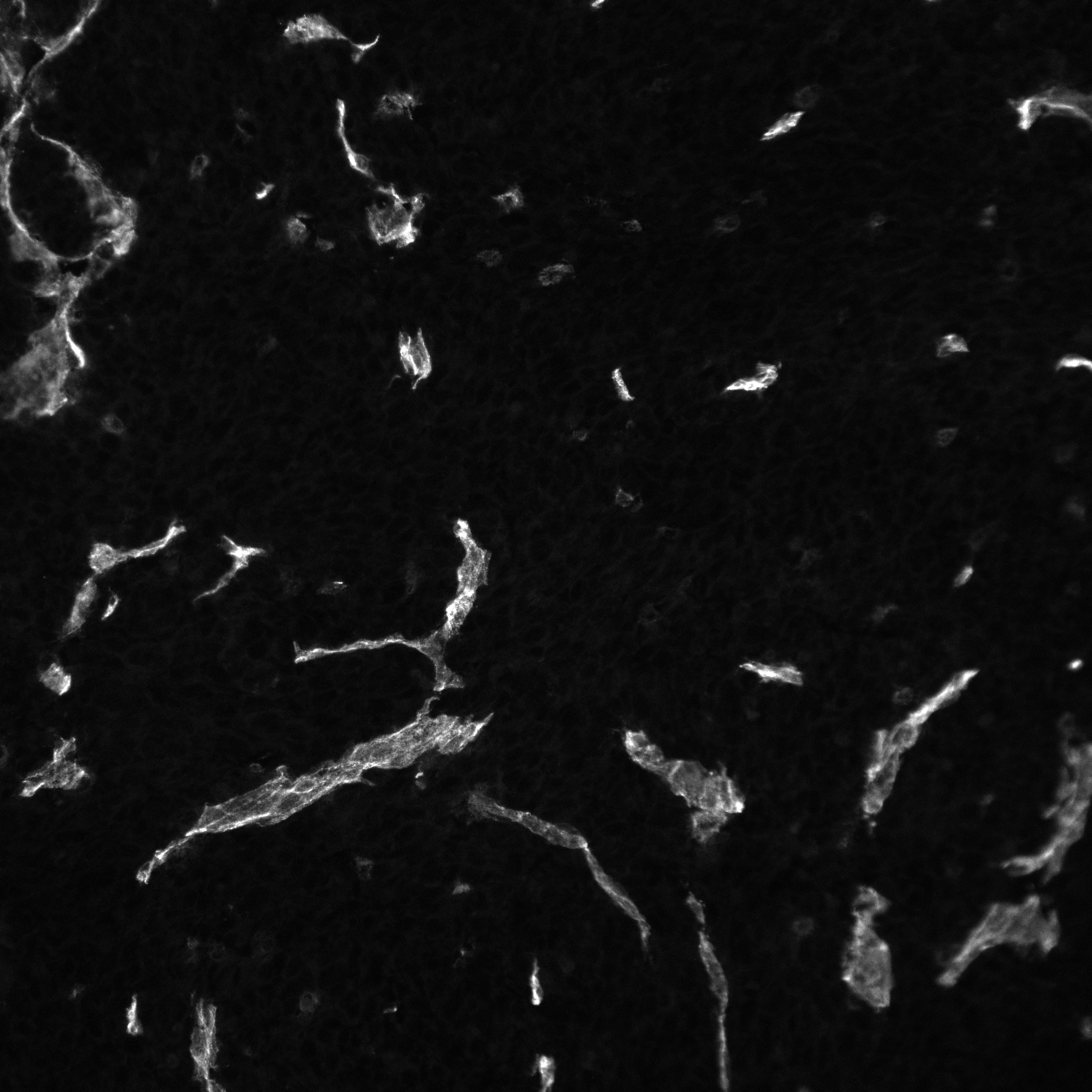

Supplement: Supplementary file 4 — Source Data Fig. 3 [file 44321_2024_34_MOESM4_ESM.zip › Figure 3/3A/Ruxolitinib_CD4_10x_bar 100┬╡m.tif]

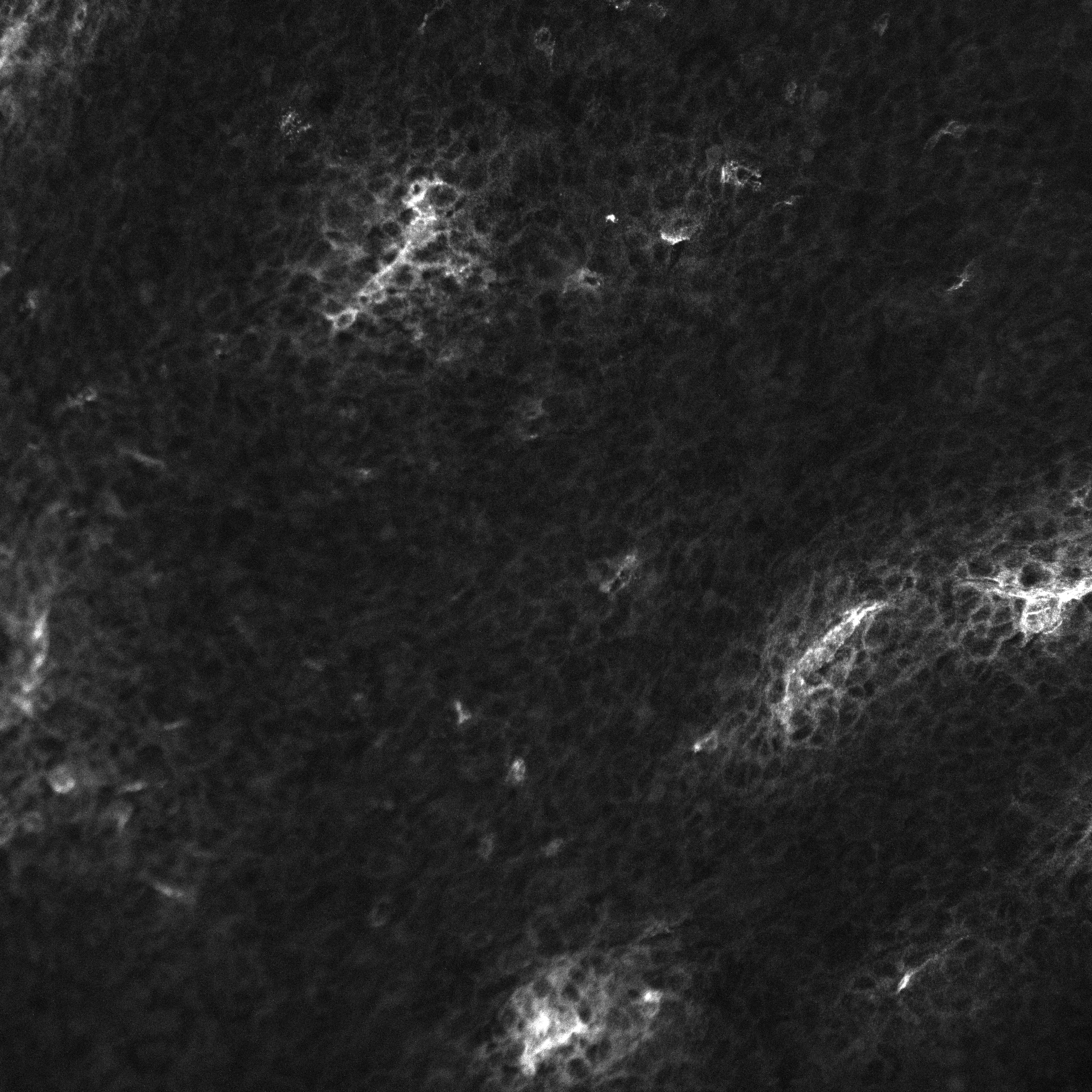

Supplement: Supplementary file 4 — Source Data Fig. 3 [file 44321_2024_34_MOESM4_ESM.zip › Figure 3/3A/Saline_CD8_10x_bar 100┬╡m.tif]

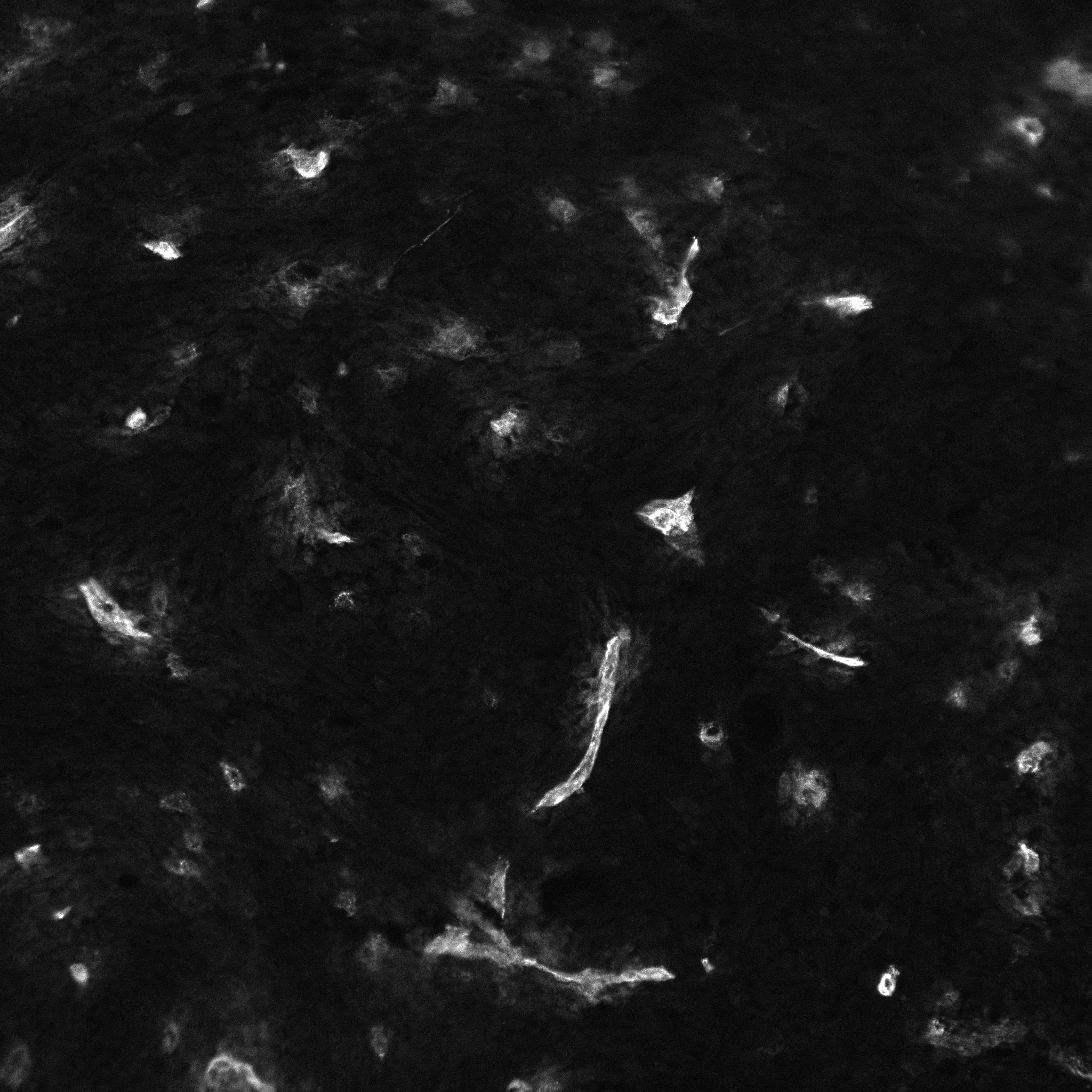

Supplement: Supplementary file 4 — Source Data Fig. 3 [file 44321_2024_34_MOESM4_ESM.zip › Figure 3/3A/L19-mIL12_CD8_10x_bar 100┬╡m.tif]

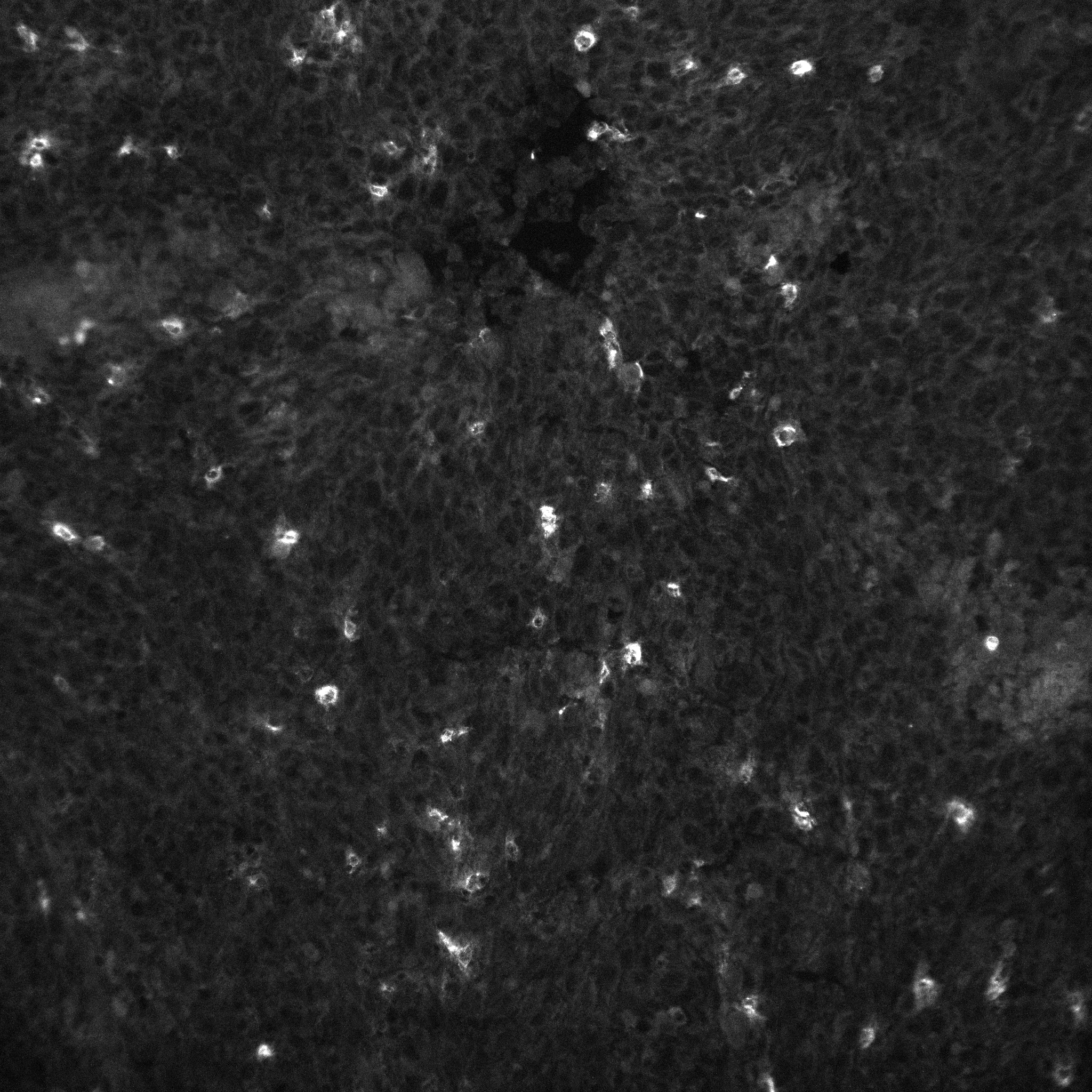

Supplement: Supplementary file 4 — Source Data Fig. 3 [file 44321_2024_34_MOESM4_ESM.zip › Figure 3/3A/Ruxo + L19-mIL12_CD4_10x_bar 100┬╡m.tif]

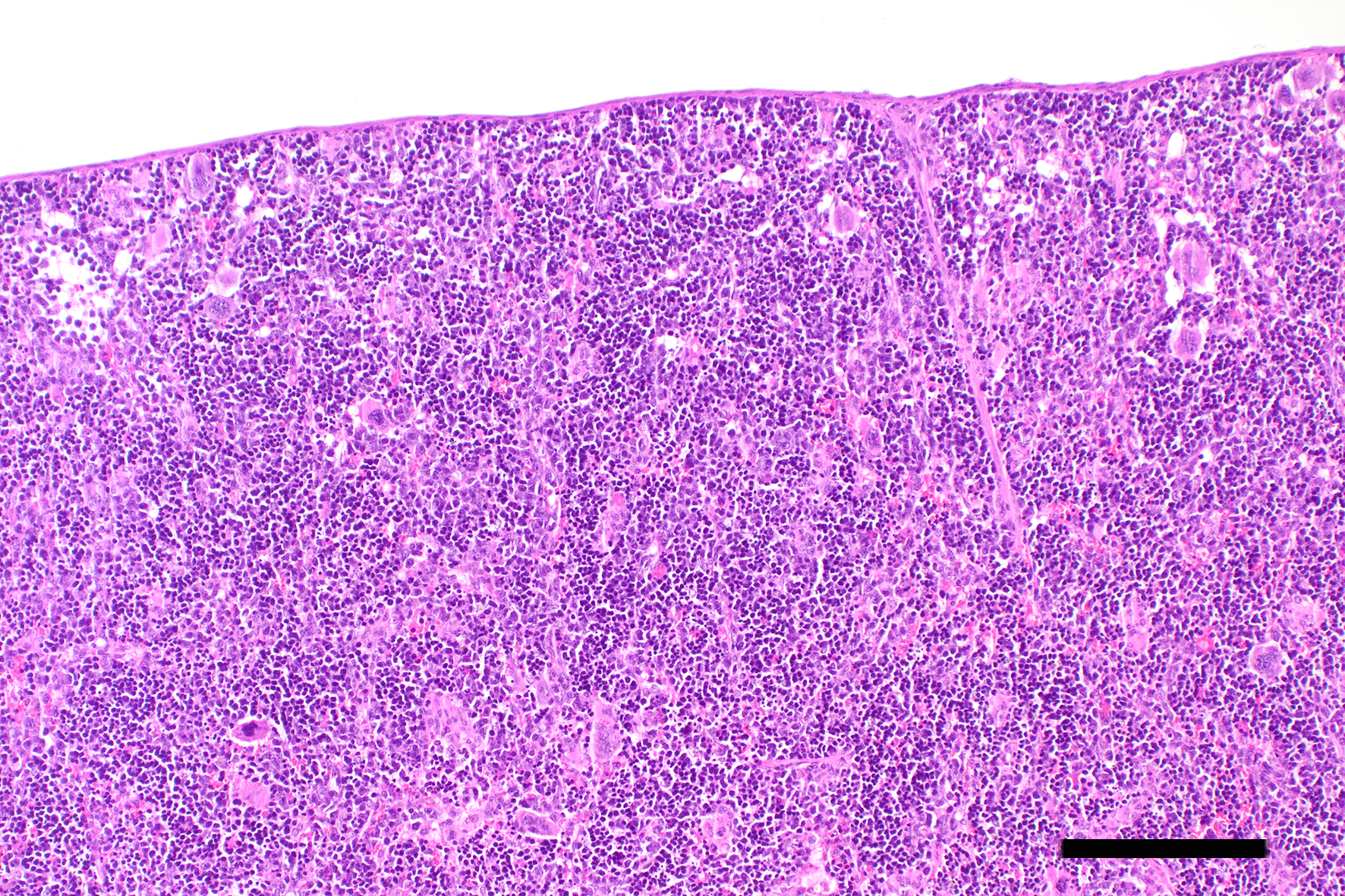

Supplement: Supplementary file 5 — Source Data Fig. 4 [file 44321_2024_34_MOESM5_ESM.zip › Figure 4/4F/L19-mIL12_spleen_bar 250┬╡m.tif]

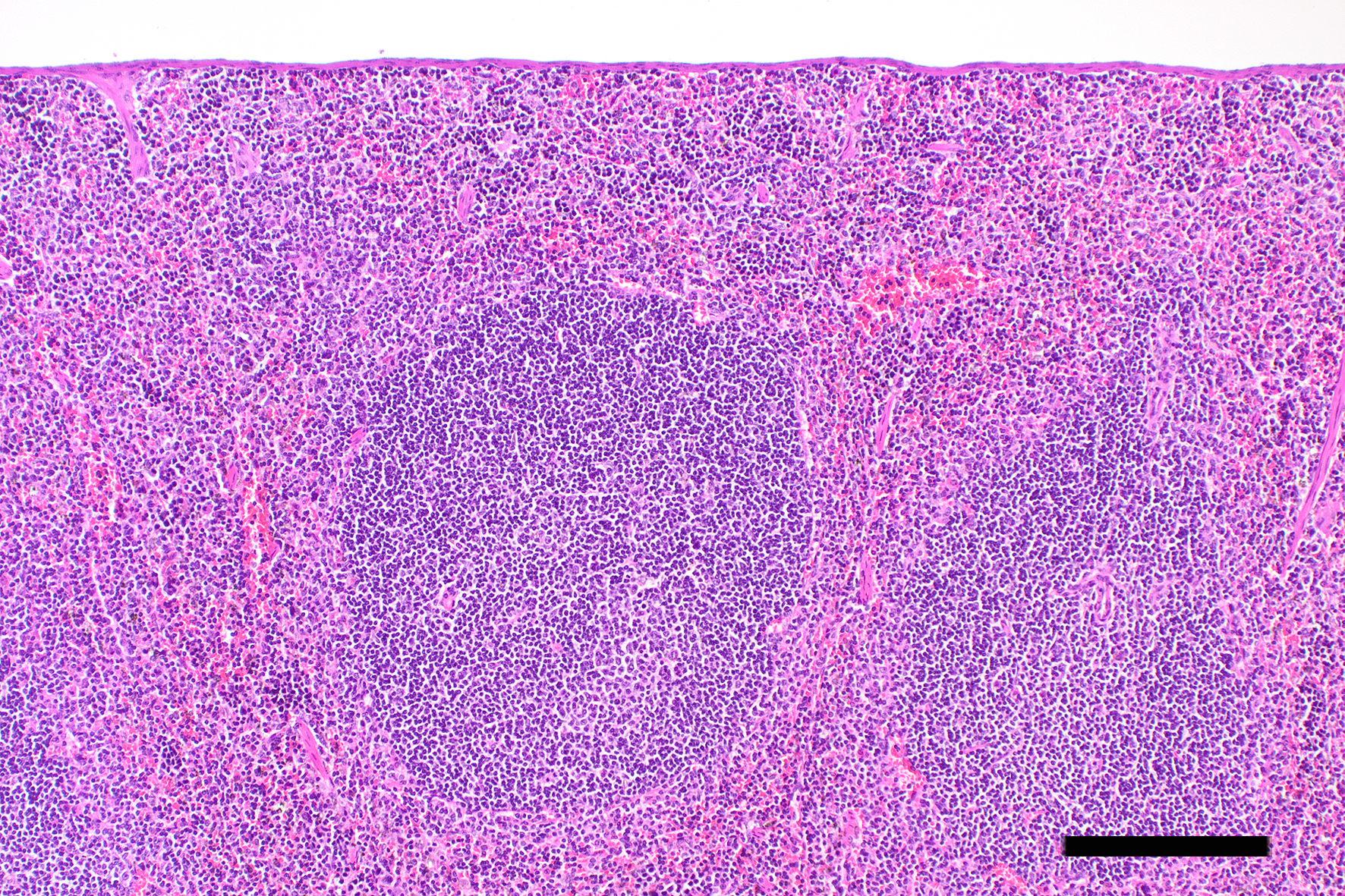

Supplement: Supplementary file 5 — Source Data Fig. 4 [file 44321_2024_34_MOESM5_ESM.zip › Figure 4/4F/Ruxolitinib_spleen_bar 250┬╡m.tif]

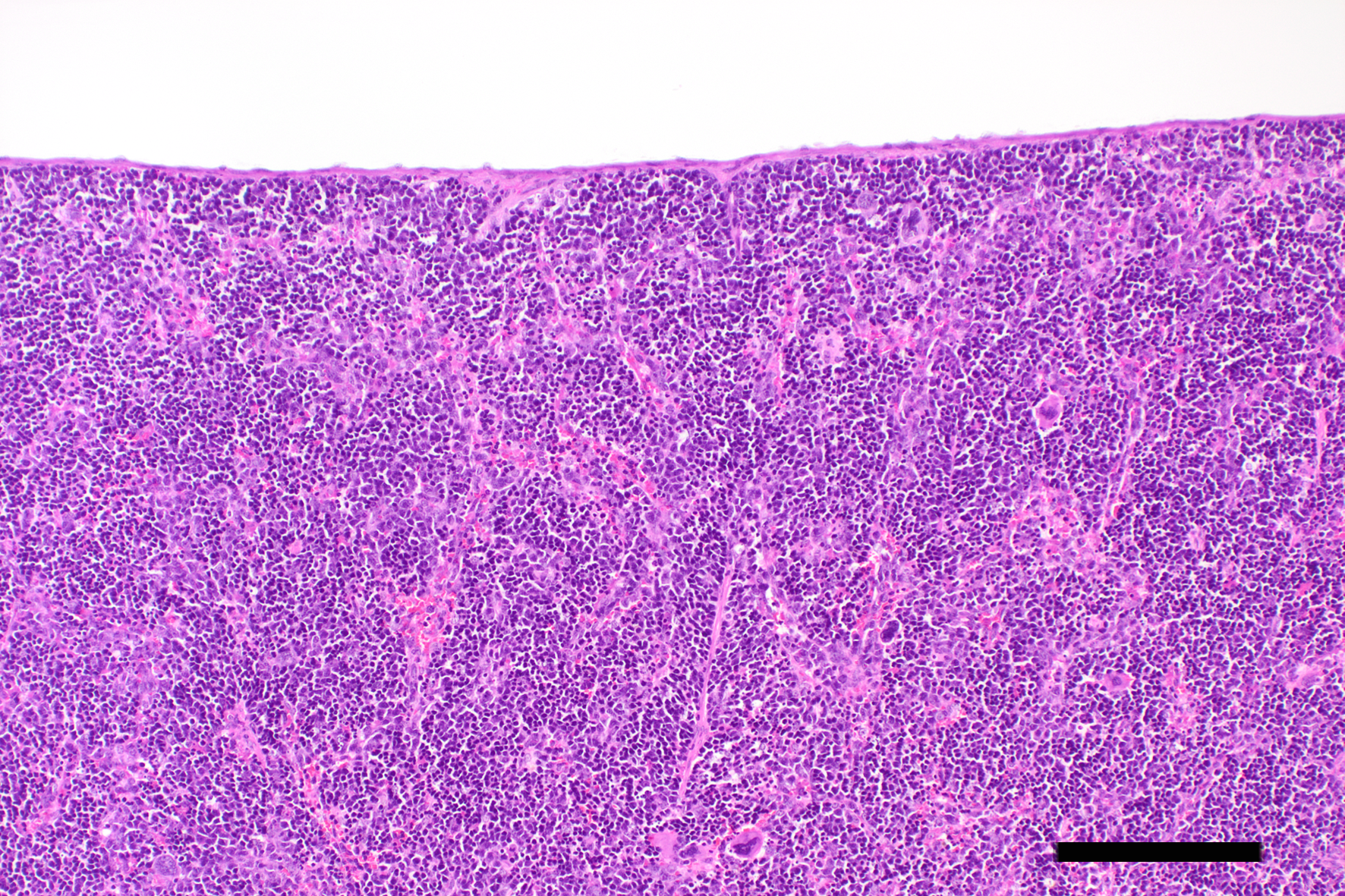

Supplement: Supplementary file 5 — Source Data Fig. 4 [file 44321_2024_34_MOESM5_ESM.zip › Figure 4/4F/Ruxo + L19-mIL12_spleen_bar 250┬╡m.tif]

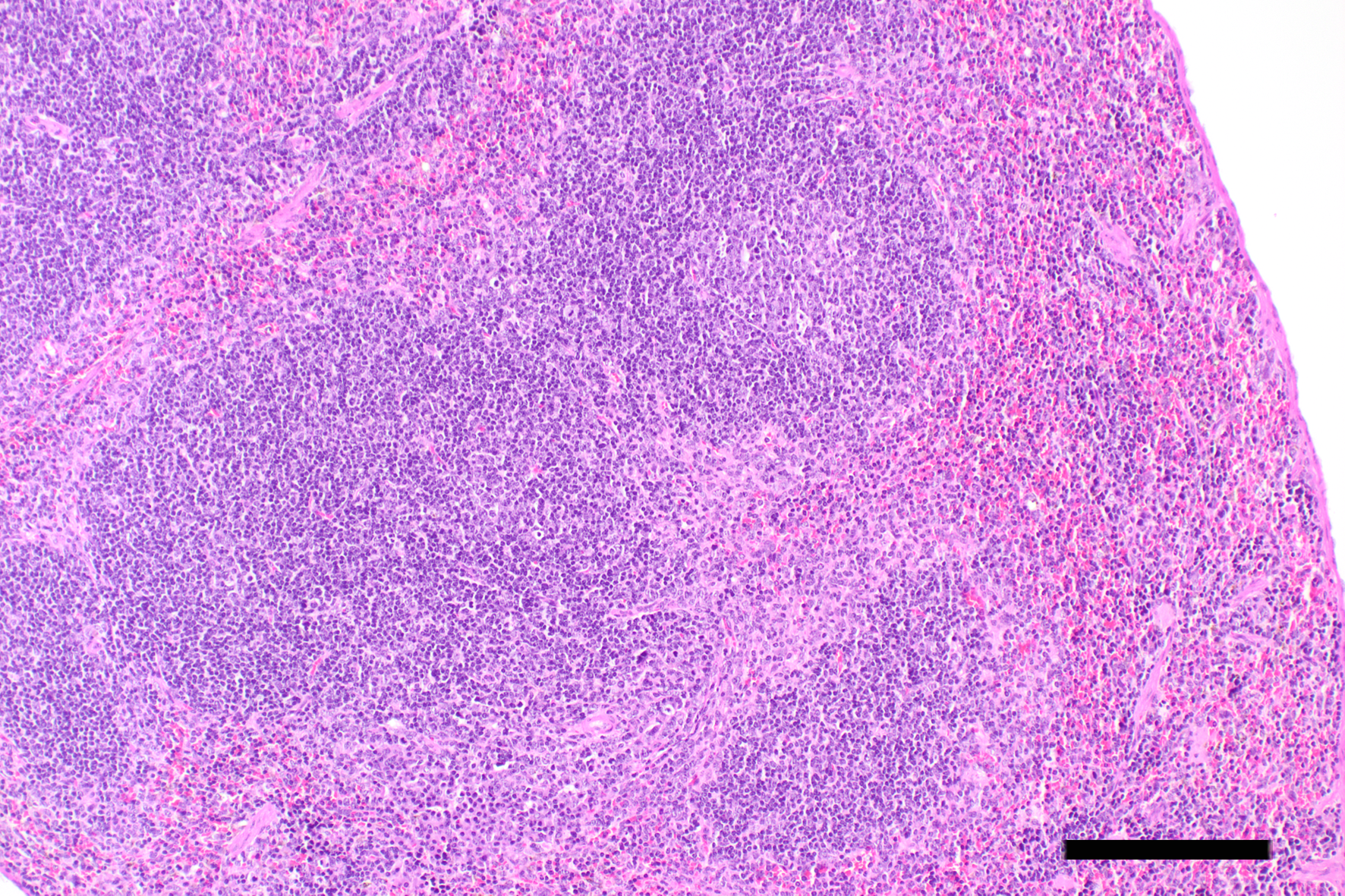

Supplement: Supplementary file 5 — Source Data Fig. 4 [file 44321_2024_34_MOESM5_ESM.zip › Figure 4/4F/Saline_spleen_bar 250┬╡m.tif]

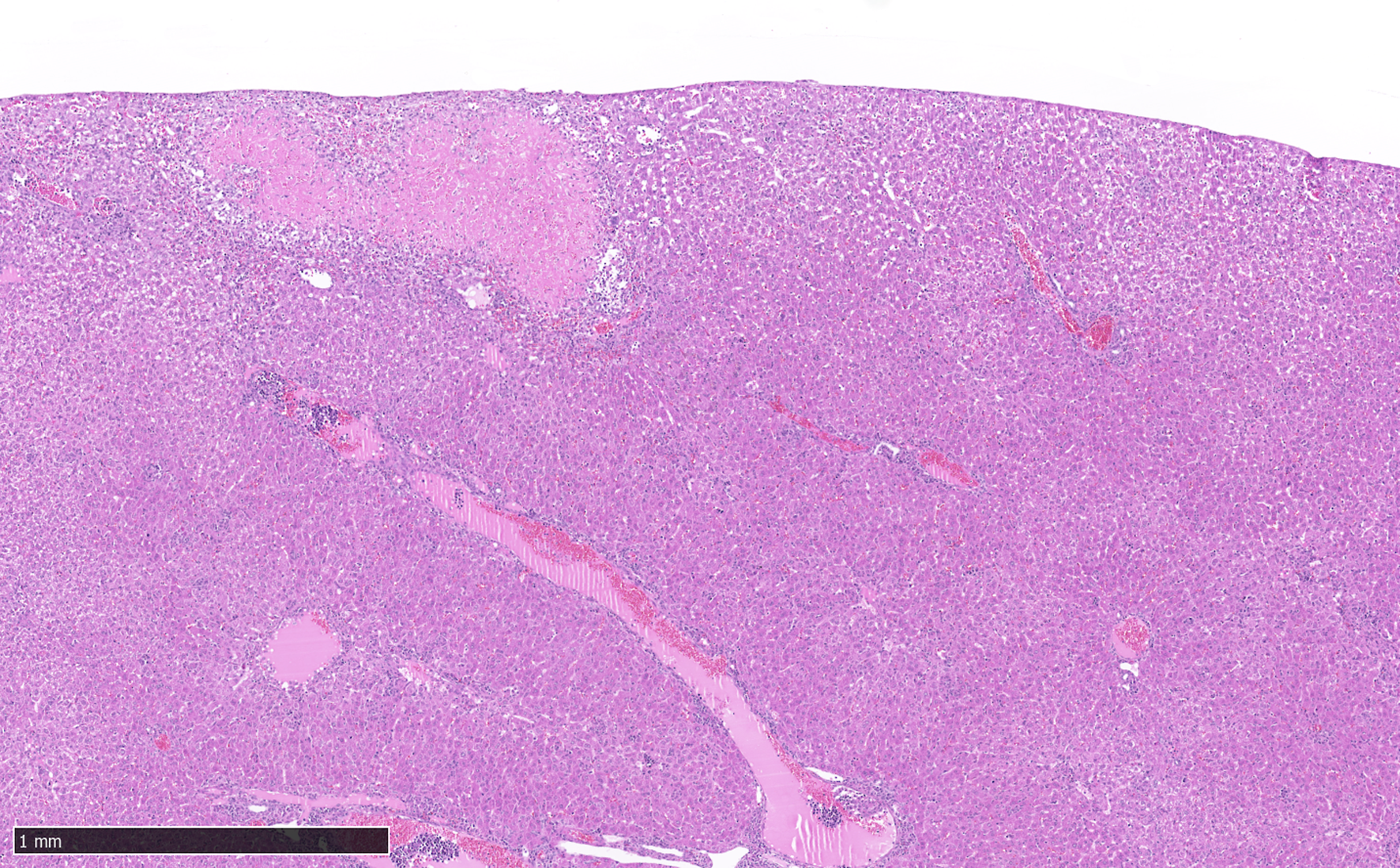

Supplement: Supplementary file 6 — Source Data Fig. 5 [file 44321_2024_34_MOESM6_ESM.zip › Figure 5/5A/L19-mIL12_overview_bar 1mm.tif]

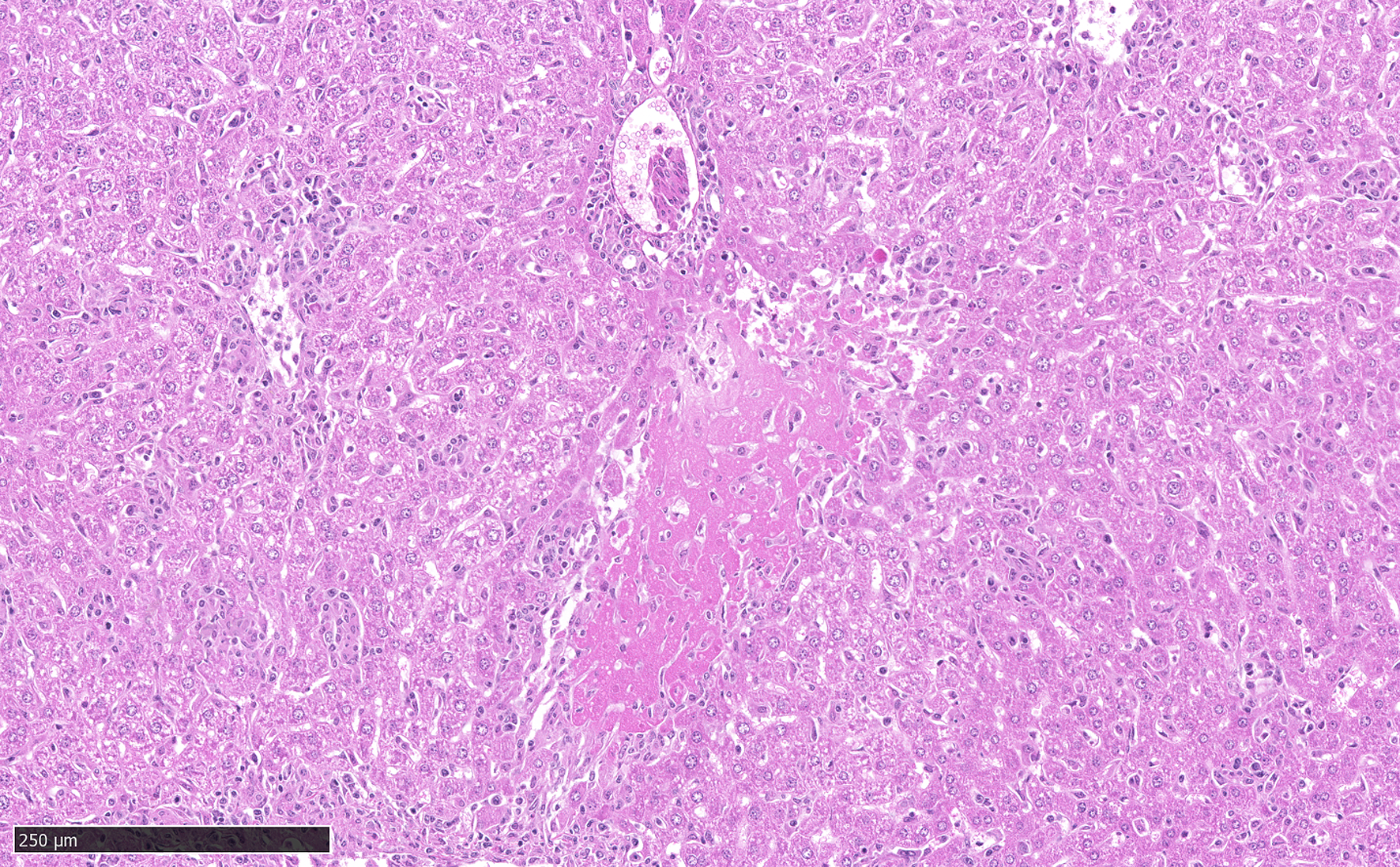

Supplement: Supplementary file 6 — Source Data Fig. 5 [file 44321_2024_34_MOESM6_ESM.zip › Figure 5/5A/Ruxo + L19-mIL12_detail_bar 250┬╡m.tif]

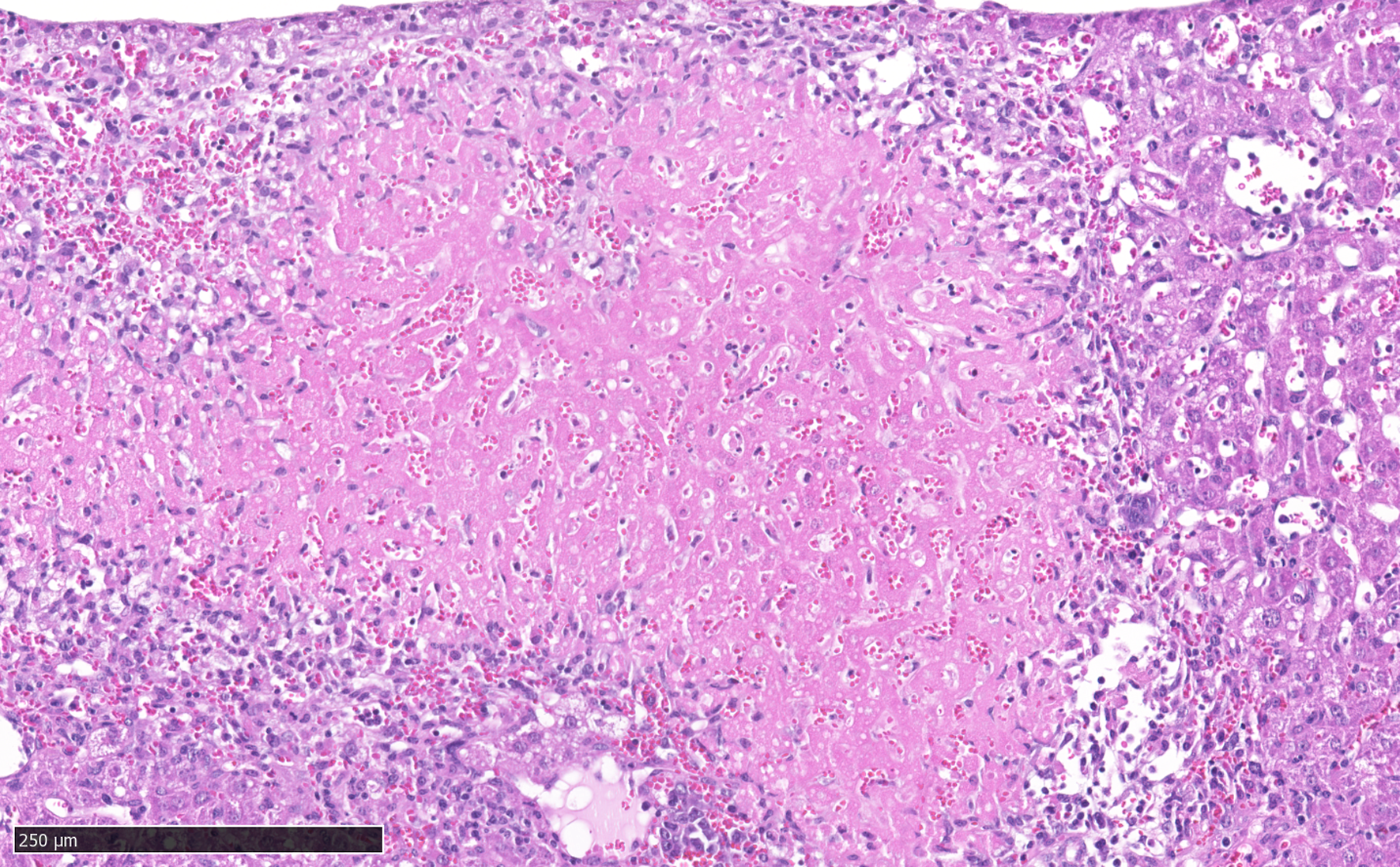

Supplement: Supplementary file 6 — Source Data Fig. 5 [file 44321_2024_34_MOESM6_ESM.zip › Figure 5/5A/L19-mIL12_detail_bar 250┬╡m.tif]

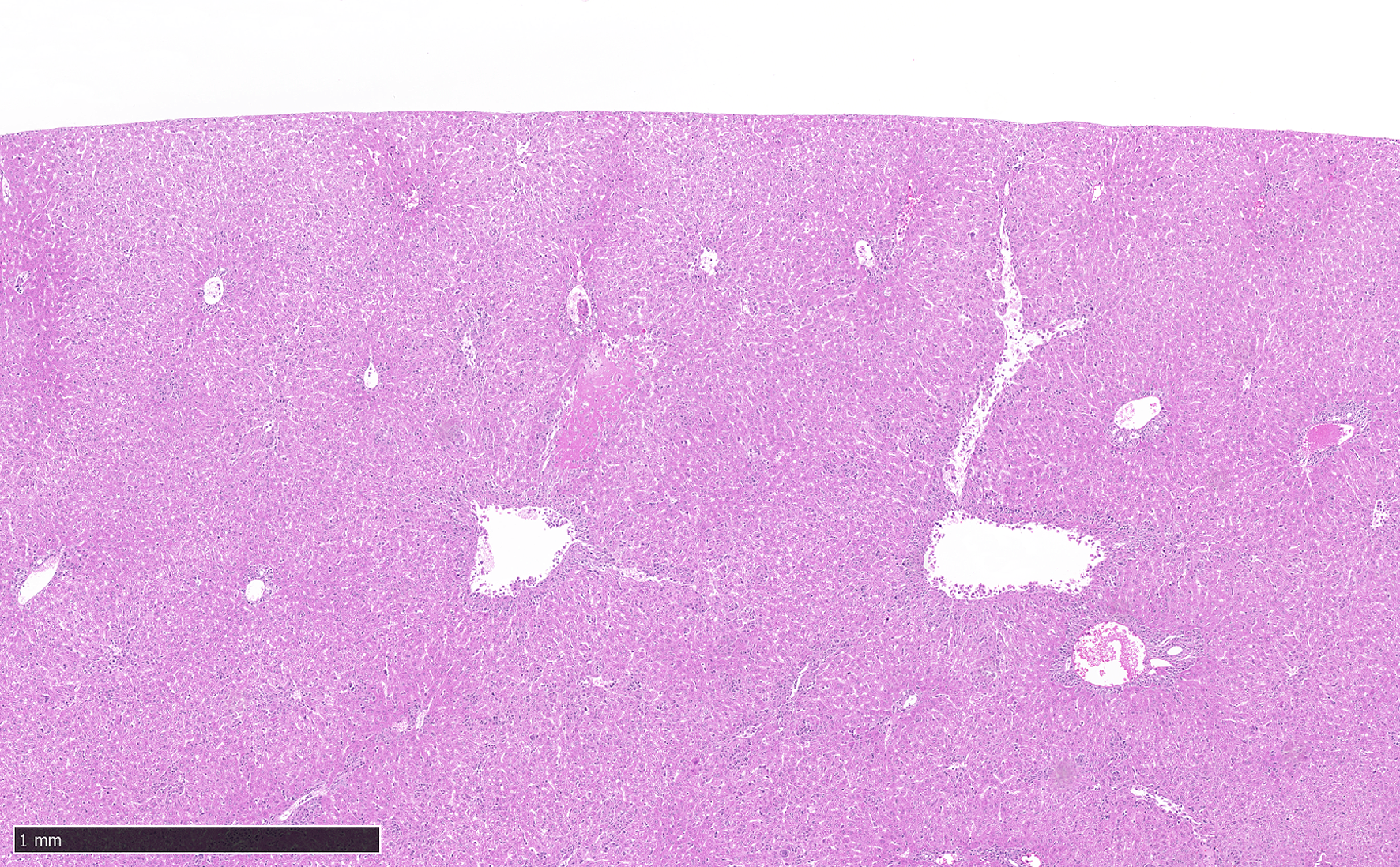

Supplement: Supplementary file 6 — Source Data Fig. 5 [file 44321_2024_34_MOESM6_ESM.zip › Figure 5/5A/Ruxo + L19-mIL12_overview_bar 1mm.tif]

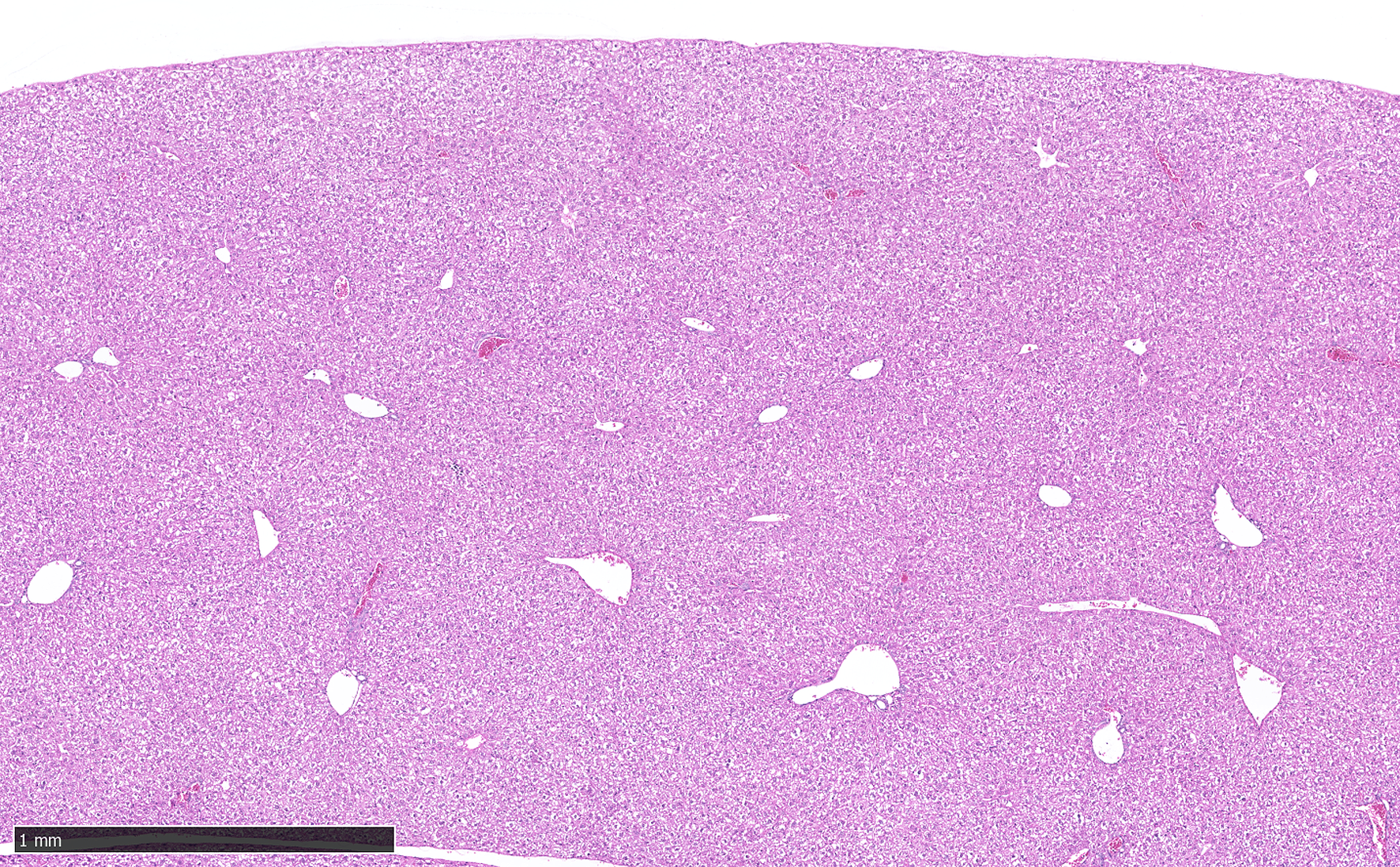

Supplement: Supplementary file 6 — Source Data Fig. 5 [file 44321_2024_34_MOESM6_ESM.zip › Figure 5/5A/Saline_overview_bar 1mm.tif]

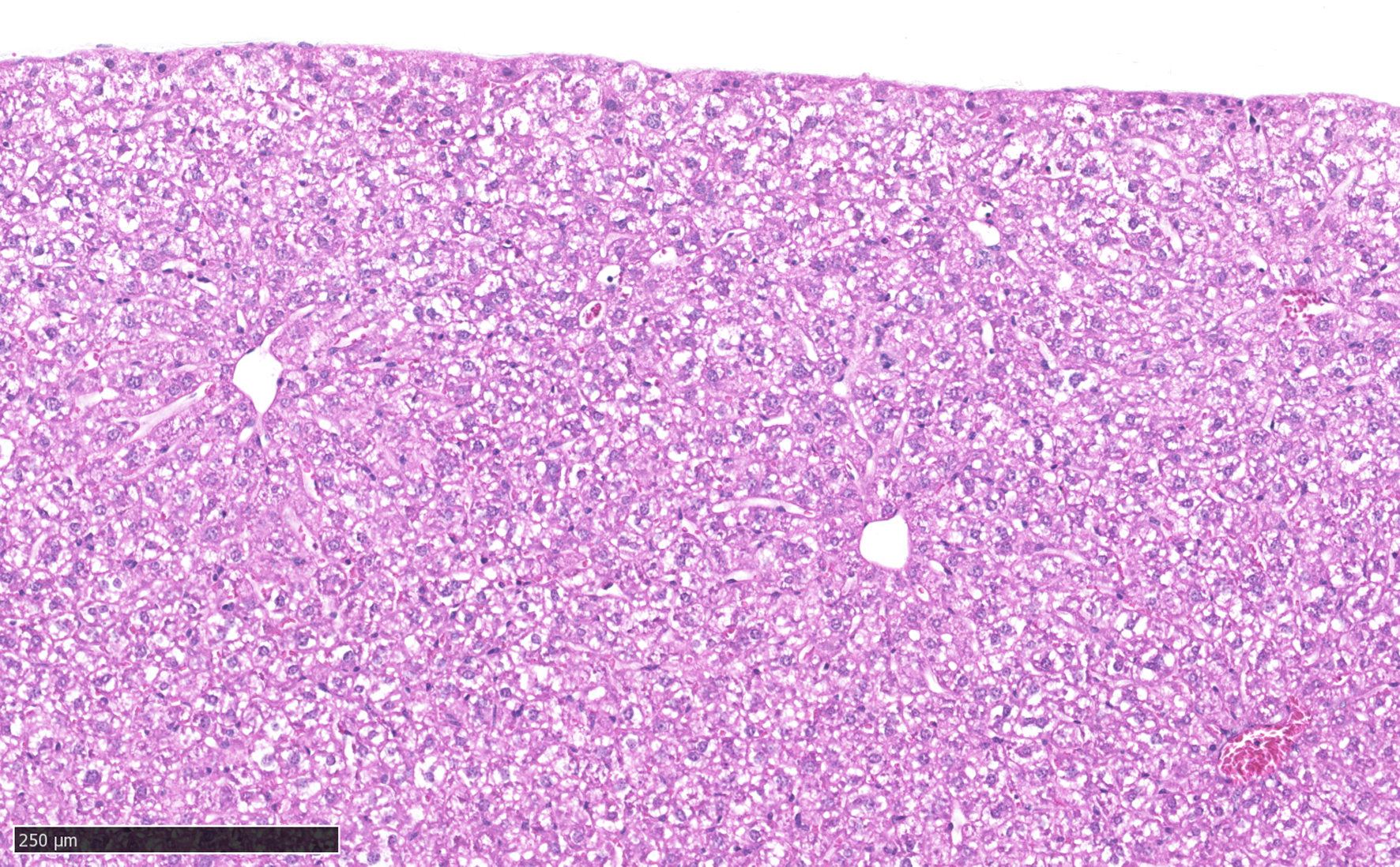

Supplement: Supplementary file 6 — Source Data Fig. 5 [file 44321_2024_34_MOESM6_ESM.zip › Figure 5/5A/Saline_detail_bar 250┬╡m.tif]

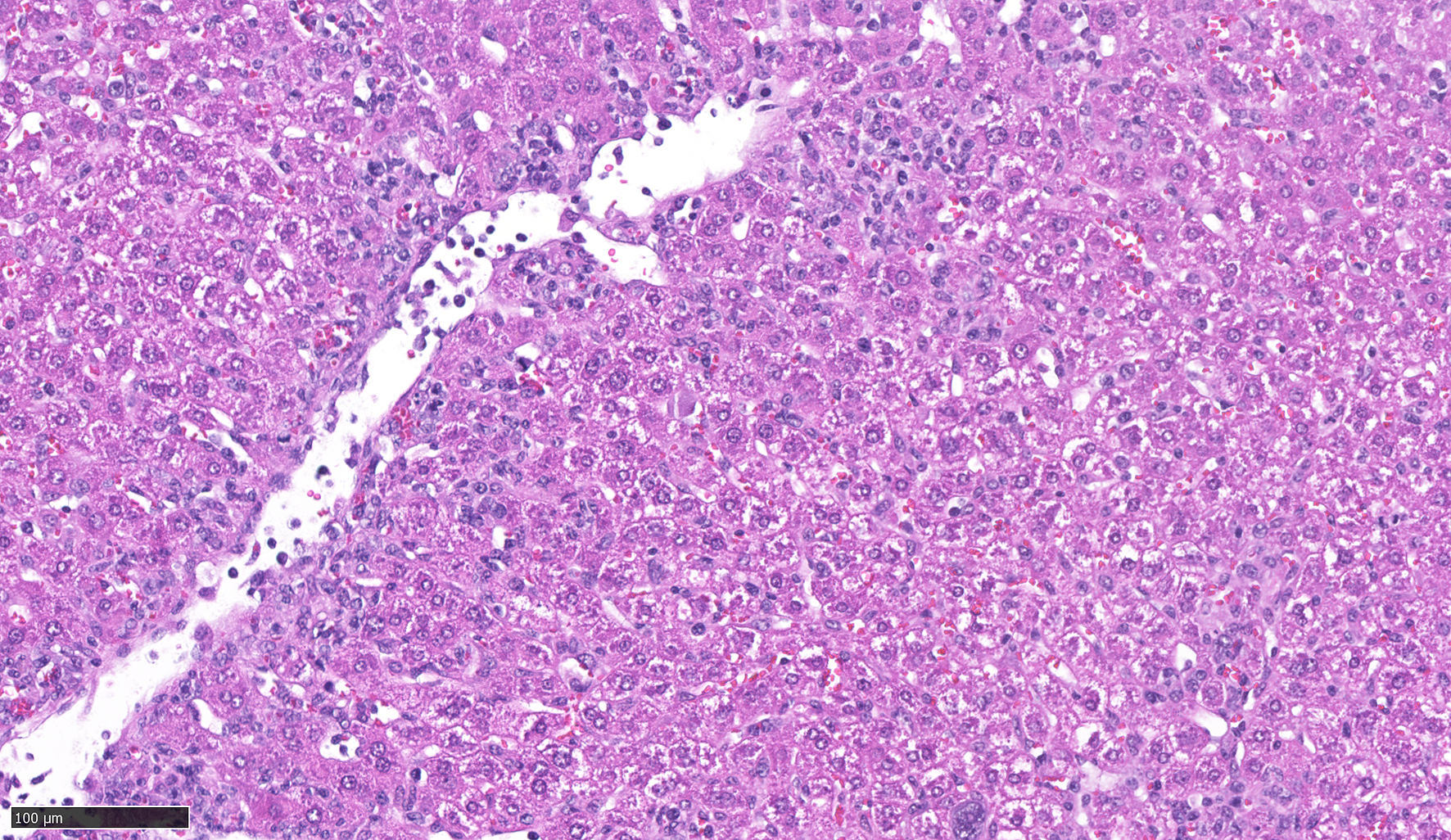

Supplement: Supplementary file 7 — Source Data Fig. 6 [file 44321_2024_34_MOESM7_ESM.zip › Figure 6/6G/Ruxo + L19-mIL12_detail_20x_bar 100┬╡m.tif]

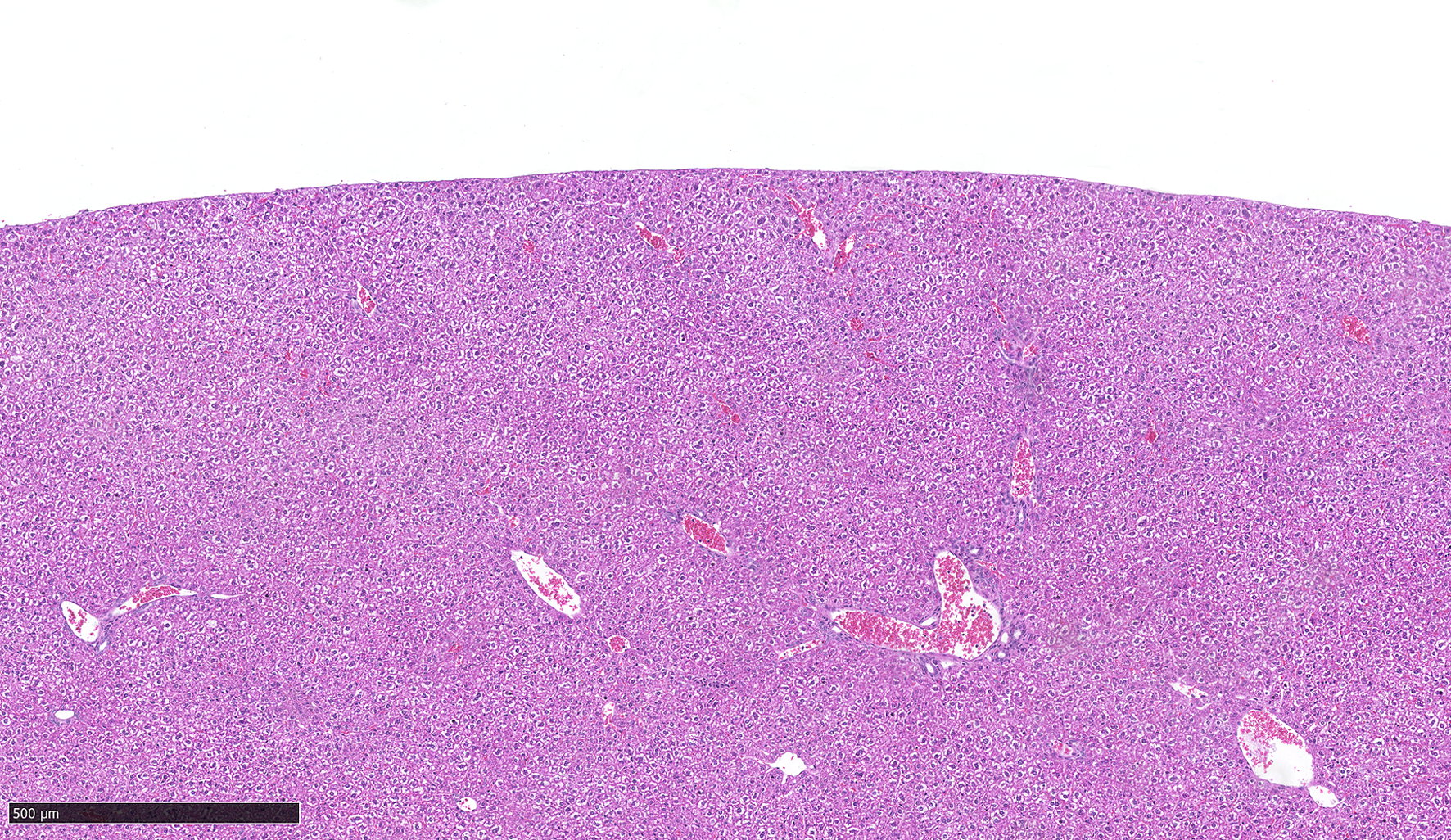

Supplement: Supplementary file 7 — Source Data Fig. 6 [file 44321_2024_34_MOESM7_ESM.zip › Figure 6/6G/Saline_overview 7x_bar 500┬╡m.tif]

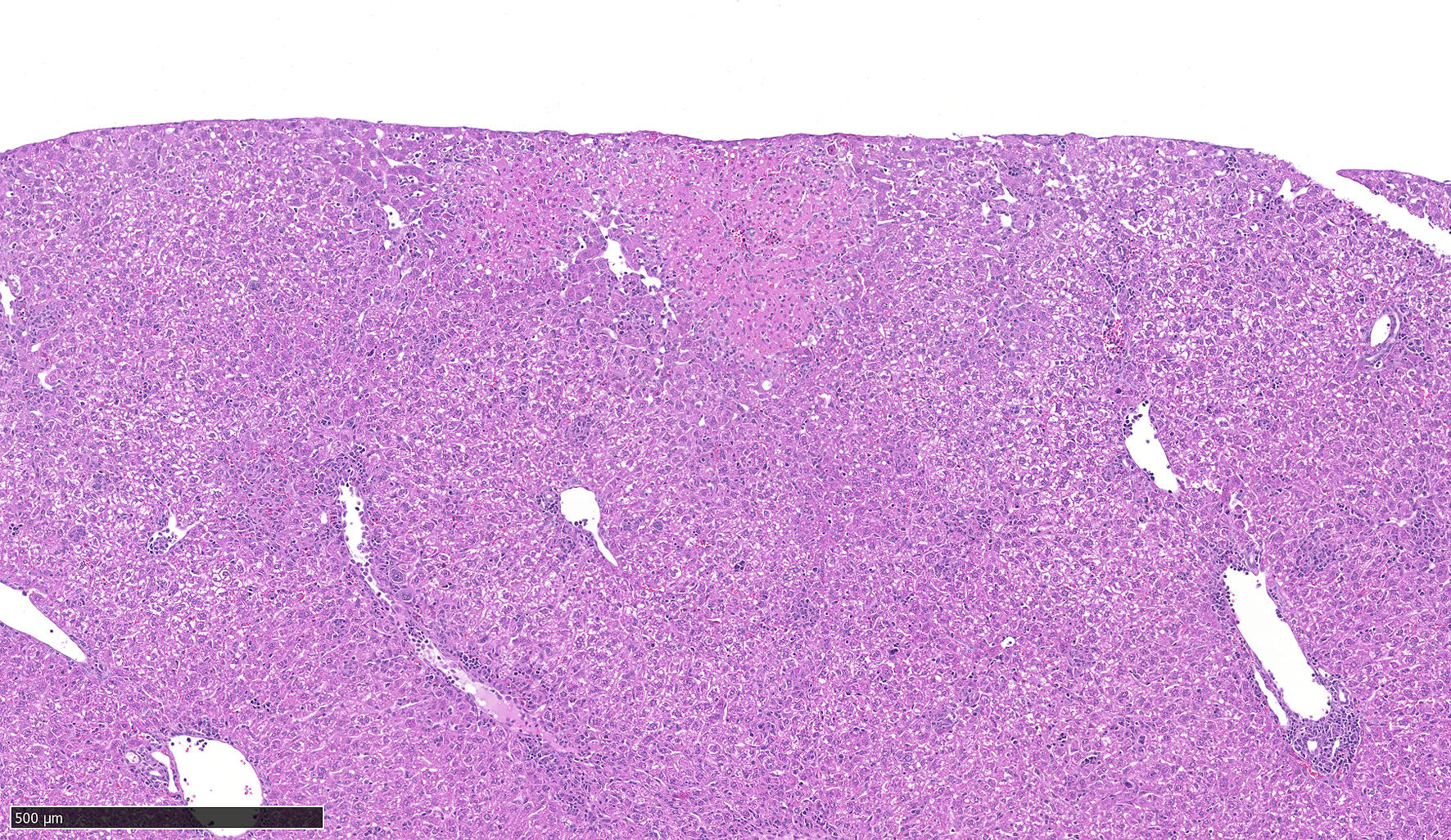

Supplement: Supplementary file 7 — Source Data Fig. 6 [file 44321_2024_34_MOESM7_ESM.zip › Figure 6/6G/L19-mIL12_overview 7x_bar 500┬╡m.tif]

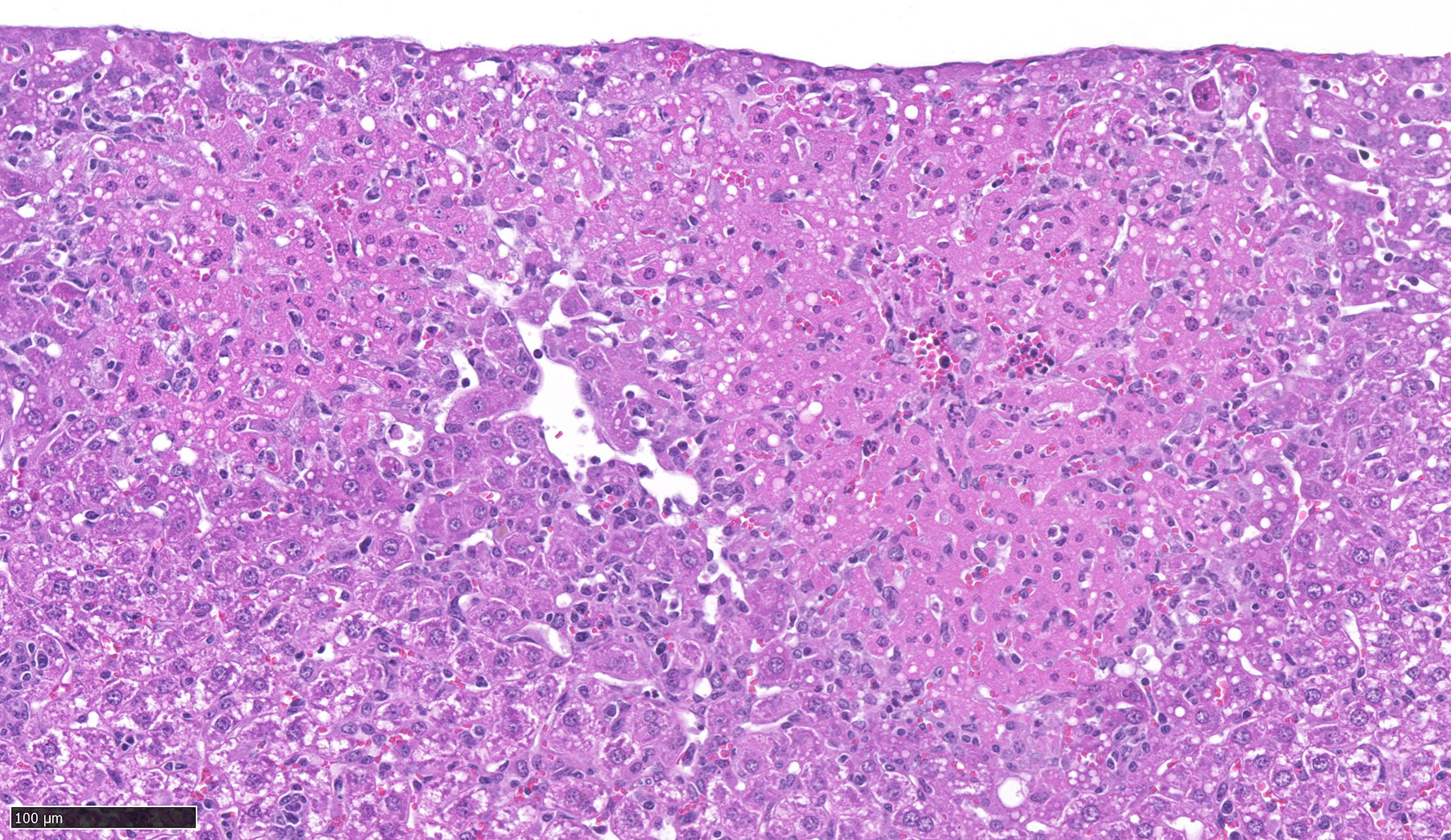

Supplement: Supplementary file 7 — Source Data Fig. 6 [file 44321_2024_34_MOESM7_ESM.zip › Figure 6/6G/L19-mIL12_detail_20x_bar 100┬╡m.tif]

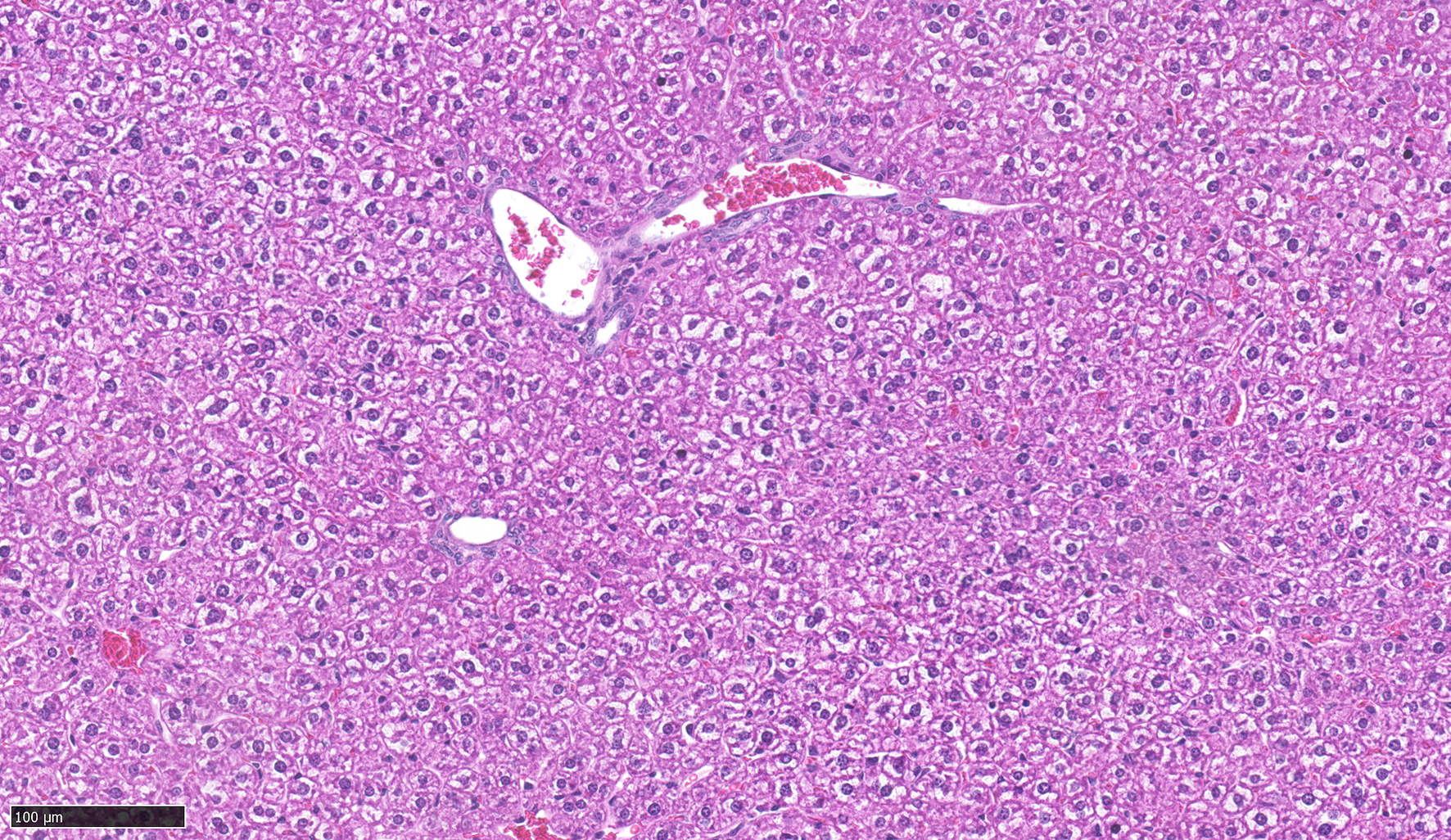

Supplement: Supplementary file 7 — Source Data Fig. 6 [file 44321_2024_34_MOESM7_ESM.zip › Figure 6/6G/Saline_detail_20x_bar 100┬╡m.tif]

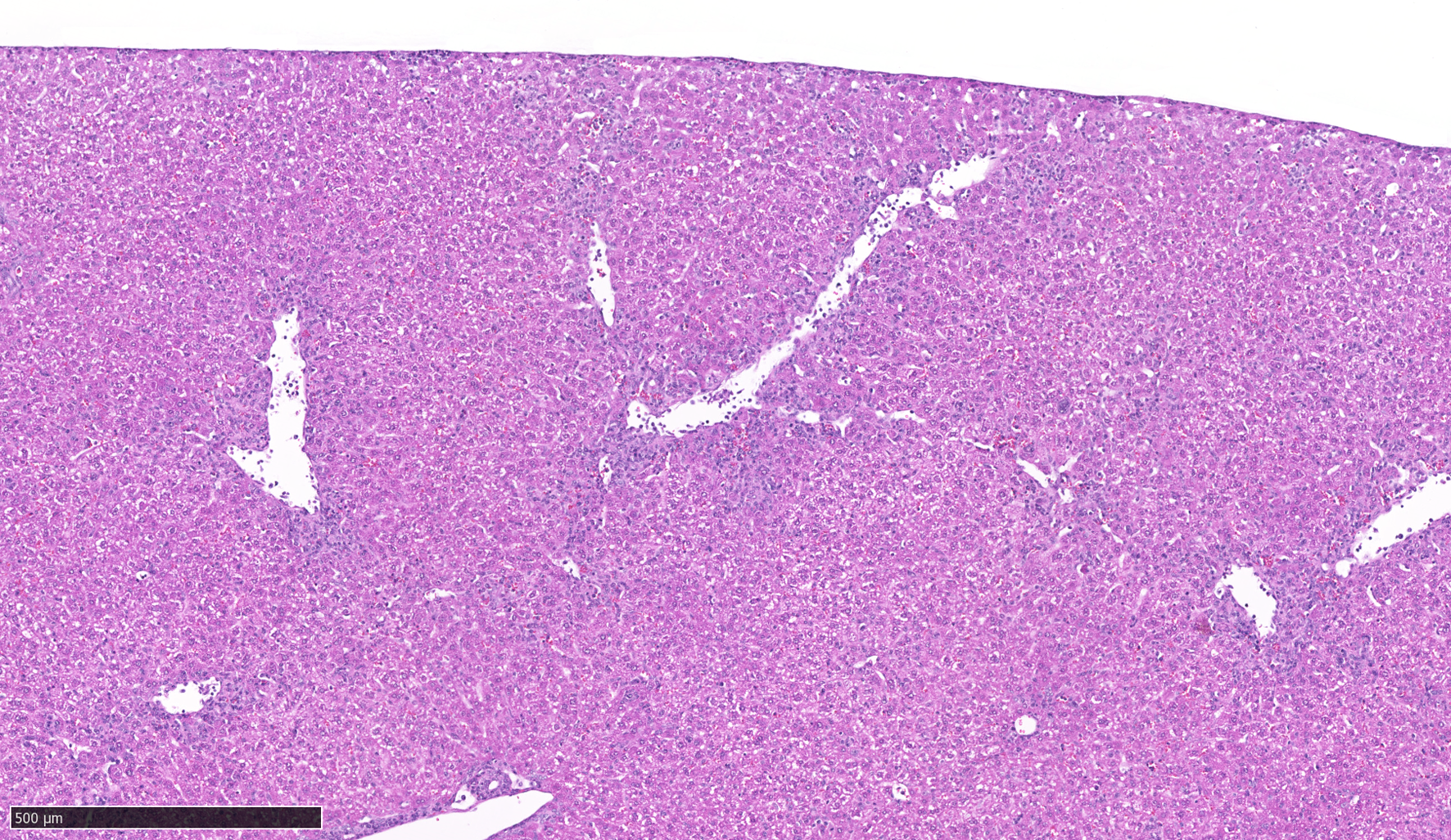

Supplement: Supplementary file 7 — Source Data Fig. 6 [file 44321_2024_34_MOESM7_ESM.zip › Figure 6/6G/Ruxo + L19-mIL12_overview 7x_bar 500┬╡m.tif]
