# Supplementary material for: A novel strategy to generate immunocytokines with activity-on-demand using small molecule inhibitors
Source: EMBO Mol Med. 2024 Mar 6;16(4):18. doi: 10.1038/s44321-024-00034-0 (PMC11018789; doi:10.1038/s44321-024-00034-0)
Supplement: Supplementary file 9 — Expanded View Figures [file 44321_2024_34_MOESM9_ESM.pdf]

Expanded View Figures

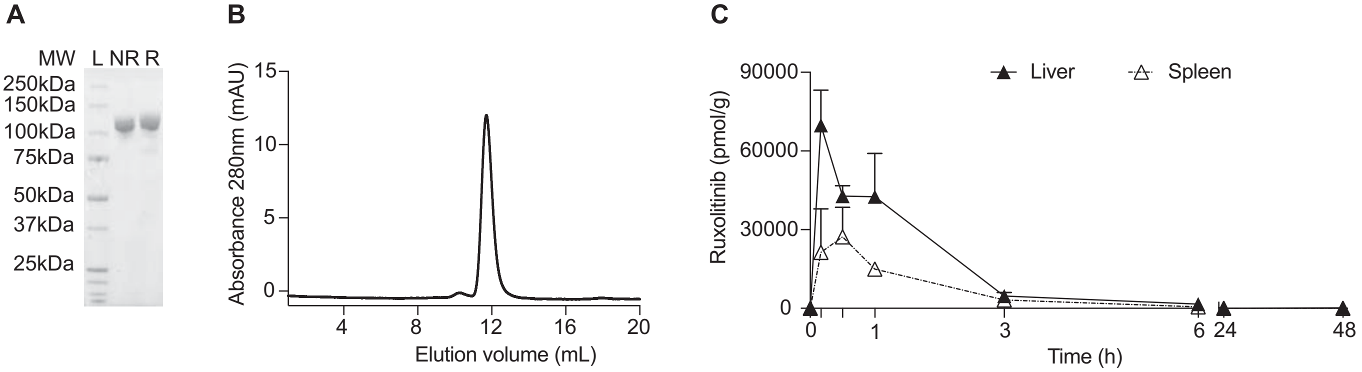

Figure EV1. Ruxolitinib exhibits an optimal pharmacokinetic profile with rapid body clearance.

(**A**,**B**) Quality control analysis of L19-IL12 fusion protein assessed by SDS-PAGE (**A**) and size exclusion chromatography (**B**). MW = molecular weight; L = ladder; NR = non-reducing conditions; R = reducing conditions. (**C**) Quantitative ex vivo MS-based biodistribution of Ruxolitinib at different time points after a single dose in MC-38 tumor-bearing mice (75 mg/kg, SC). Results are expressed as picomoles per gram of tissue (mean ± SD, $n = 3$ mice per time point).

 © The Author(s)

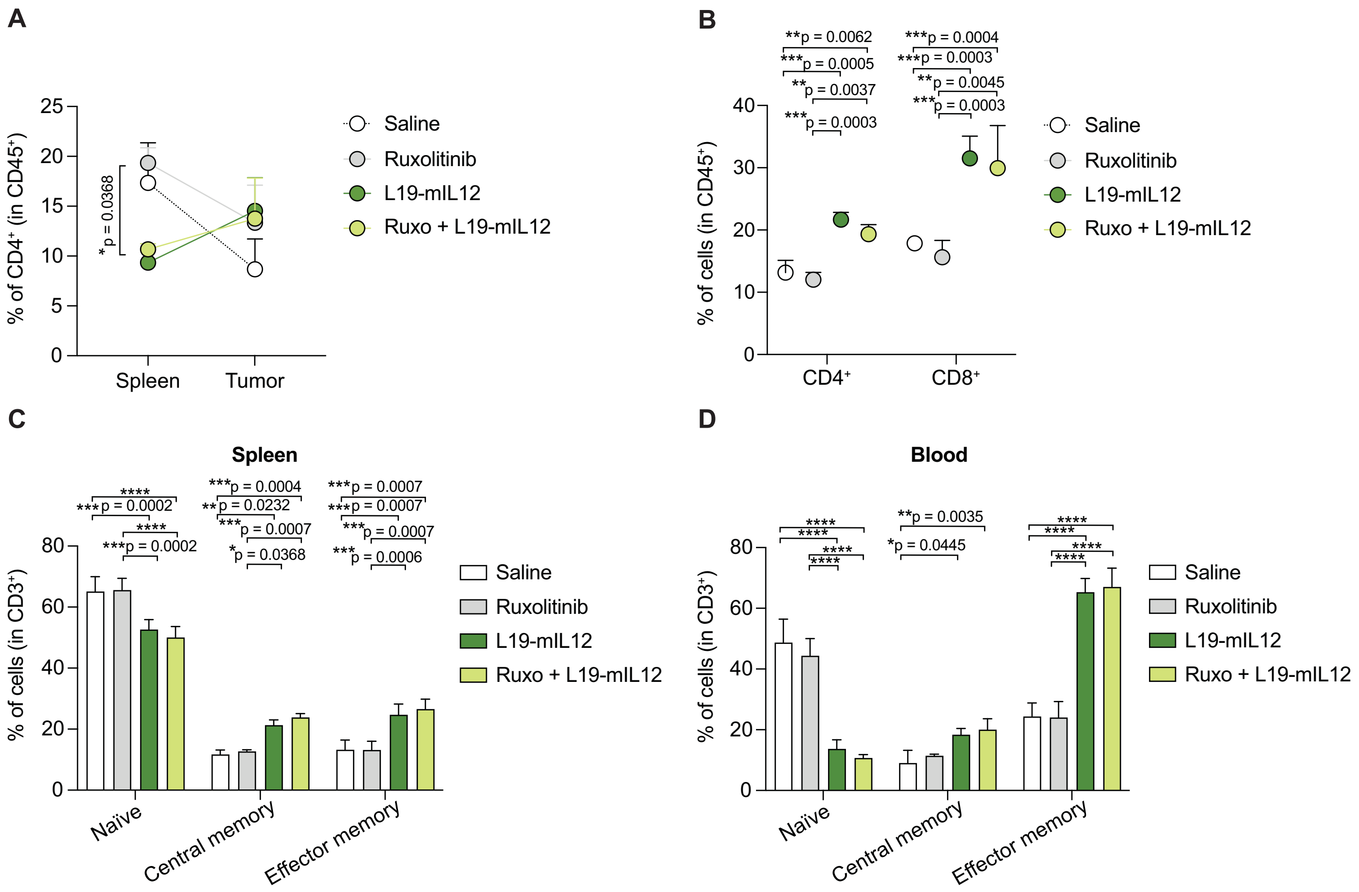

Figure EV2. Pre-treatment with Ruxolitinib preserves the immunological remodeling induced by L19-mIL12 in blood and spleen.

MC-38 tumor-bearing mice were euthanized 24 h after the third injection of either saline, Ruxolitinib (75 mg/kg, s.c), L19-mIL12 (1.2 mg/kg, i.v), or Ruxolitinib pre-administered before the L19-mIL12 ($n = 3$–5 mice per group). (**A–D**) Percentage of CD4 + T cells among CD45+ cells in tumors and spleens (**A**), percentage of CD8+ and CD4 + T cells among CD45+ cells in blood (**B**). Percentage of Naïve (CD44$^-$CD62L$^+$), Central Memory (CD44$^+$CD62L$^+$), and Effector Memory (CD44$^+$CD62L$^-$) cells among CD3+ cells in the spleen (**C**) and blood (**D**). Data information: in (**A–D**), data represent mean ± SD. One-way ANOVA analysis (*$p < 0.05$; **$p < 0.01$; ***$p < 0.001$; ****$p < 0.0001$).

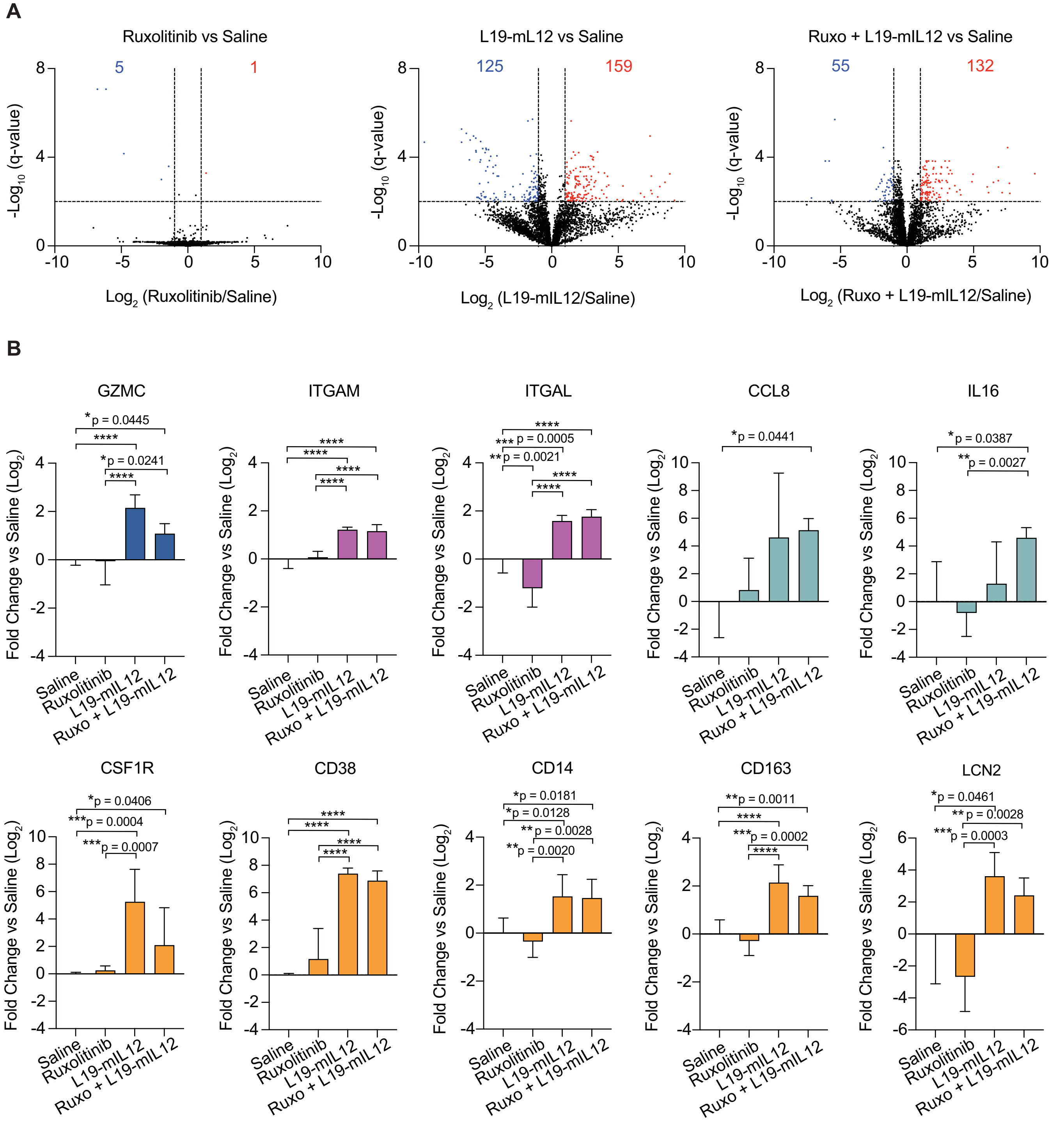

Figure EV3. Pre-treatment with Ruxolitinib preserves the remodeling of the tumor proteome induced by L19-mIL12.

MC-38 tumor-bearing mice were euthanized 24 h after the third injection of either saline, Ruxolitinib (75 mg/kg, s.c), or L19-mIL12 (1.2 mg/kg, i.v) alone or in combination with a pre-treatment of Ruxolitinib ($n = 3$ mice per group). (**A**) Volcano plot representation of the proteomic changes in Ruxolitinib, L19-mIL12 monotherapy or combination, compared to the saline group. Red and blue dots represent significantly up- and down-regulated proteins with FDR < 0.01 and magnitude of change >2-fold. (**B**) Expression of granzymes (blue), integrins (violet), cytokines (light blue), and immune cell markers (orange) represented as fold change compared to the saline group ($n = 3$ mice per group). Data represent mean ± SD. One-way ANOVA analysis (*$p < 0.05$; **$p < 0.01$; ***$p < 0.001$; ****$p < 0.0001$).

© The Author(s)

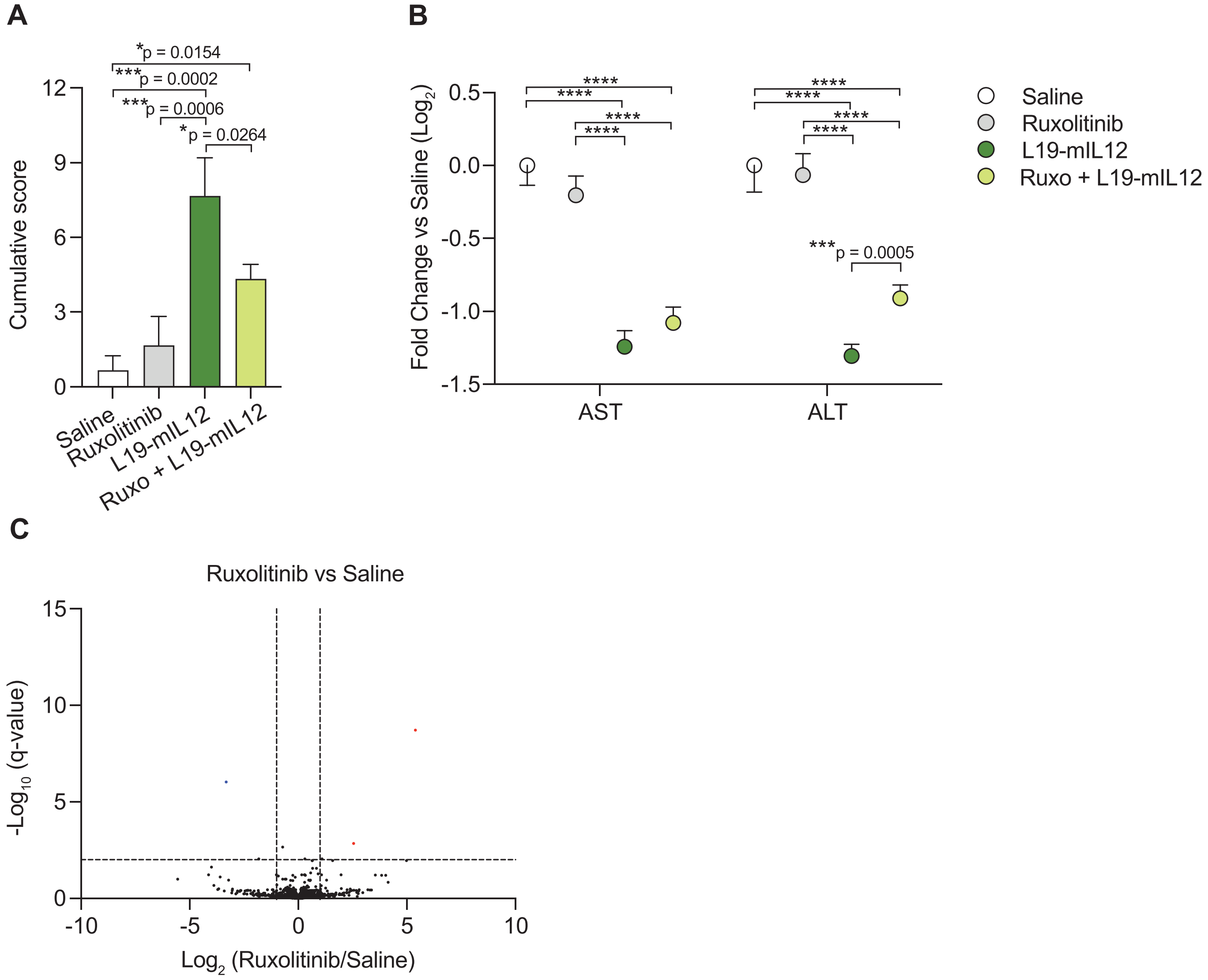

Figure EV4. Pre-treatment with Ruxolitinib reduces the hepatic changes associated with high doses of L19-mIL12.

MC-38 tumor-bearing mice were euthanized 24 h after the third injection of either saline, Ruxolitinib (75 mg/kg, s.c), or L19-mIL12 (1.2 mg/kg, i.v) alone or in combination with a pre-treatment of Ruxolitinib ($n = 3$ mice per group). (**A**) Quantification of liver damage is described as a cumulative score, including coagulation necrosis, single cell necrosis, periportal infiltrates, lobular infiltrates, and vacuolar changes in hepatocytes. (**B**) Levels of aspartate aminotransferase (AST) and alanine aminotransferase (ALT) in liver tissues. (**C**) Volcano plot representation of the liver proteomic changes in the Ruxolitinib group compared to the saline group. Red and blue dots represent significantly up- and down-regulated proteins with FDR < 0.01 and magnitude of change >2-fold. Data information: in (**A**,**B**), data represent mean ± SD. One-way ANOVA analysis (*$p < 0.05$; ***$p < 0.001$; ****$p < 0.0001$).

© The Author(s)

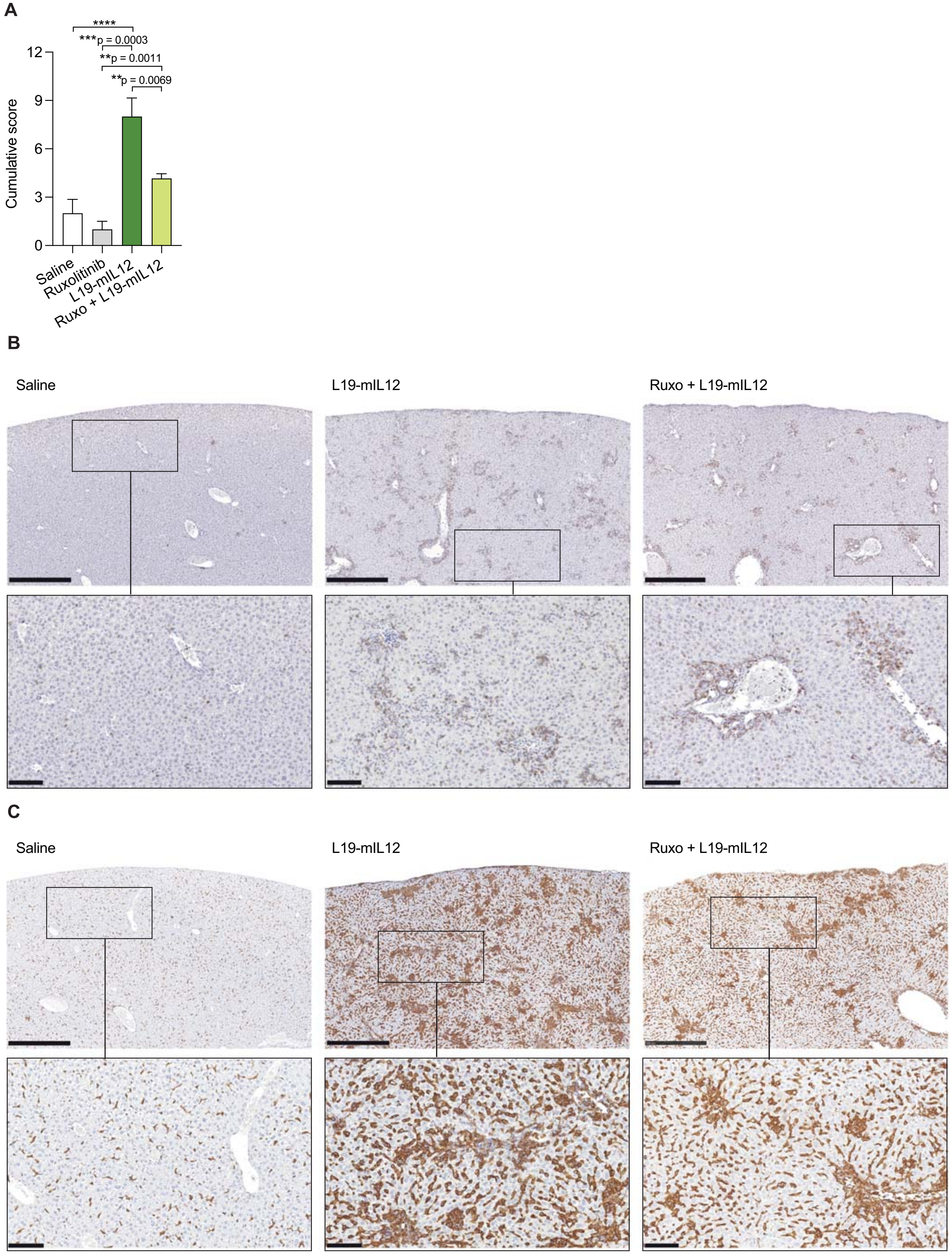
A
****
***p = 0.0003
**p = 0.0011
**p = 0.0069
Cumulative score
12
9
6
3
0
Saline
Ruxolitinib
L19-mIL12
Ruxo + L19-mIL12
B
Saline
L19-mIL12
Ruxo + L19-mIL12
C
Saline
L19-mIL12
Ruxo + L19-mIL12

 © The Author(s)

Figure EV5. Schedule optimization completely abrogates the hepatotoxicity associated with high doses of L19-mIL12.

MC-38 tumor-bearing mice were euthanized 24 h after the third injection of either saline, Ruxolitinib (75 mg/kg, s.c), or L19-mIL12 (1.2 mg/kg, i.v) alone or in combination with Ruxolitinib 10 min before and 6 h after ($n = 3$ mice per group). (**A**) Quantification of liver damage is described as a cumulative score, including coagulation necrosis, single cell necrosis, periportal infiltrates, lobular infiltrates, and vacuolar changes in hepatocytes. Data represent mean ± SD. One-way ANOVA analysis (**$p < 0.01$; ***$p < 0.001$; ****$p < 0.0001$). (**B**,**C**) Immunohistochemical staining of liver sections for CD3 (**B**) and Iba1 (**C**) at 10× magnification (scale bars = 500 μm; upper panels) and 20× magnification (scale bars = 100 μm; lower panels).

© The Author(s)
